# Eco-Evolutionary Drivers of Vibrio parahaemolyticus Sequence Type 3 Expansion: Retrospective Machine Learning Approach

**DOI:** 10.2196/62747

**Published:** 2024-11-28

**Authors:** Amy Marie Campbell, Chris Hauton, Ronny van Aerle, Jaime Martinez-Urtaza

**Affiliations:** 1 School of Ocean and Earth Science University of Southampton Southampton United Kingdom; 2 Centre for Environment, Fisheries and Aquaculture Science (CEFAS) Weymouth United Kingdom; 3 Department of Genetics and Microbiology Autonomous University of Barcelona Barcelona Spain

**Keywords:** pathogen expansion, climate change, machine learning, ecology, evolution, vibrio parahaemolyticus, sequencing, sequence type 3, VpST3, genomics

## Abstract

**Background:**

Environmentally sensitive pathogens exhibit ecological and evolutionary responses to climate change that result in the emergence and global expansion of well-adapted variants. It is imperative to understand the mechanisms that facilitate pathogen emergence and expansion, as well as the drivers behind the mechanisms, to understand and prepare for future pandemic expansions.

**Objective:**

The unique, rapid, global expansion of a clonal complex of *Vibrio parahaemolyticus* (a marine bacterium causing gastroenteritis infections) named *Vibrio parahaemolyticus* sequence type 3 (VpST3) provides an opportunity to explore the eco-evolutionary drivers of pathogen expansion.

**Methods:**

The global expansion of VpST3 was reconstructed using VpST3 genomes, which were then classified into metrics characterizing the stages of this expansion process, indicative of the stages of emergence and establishment. We used machine learning, specifically a random forest classifier, to test a range of ecological and evolutionary drivers for their potential in predicting VpST3 expansion dynamics.

**Results:**

We identified a range of evolutionary features, including mutations in the core genome and accessory gene presence, associated with expansion dynamics. A range of random forest classifier approaches were tested to predict expansion classification metrics for each genome. The highest predictive accuracies (ranging from 0.722 to 0.967) were achieved for models using a combined eco-evolutionary approach. While population structure and the difference between introduced and established isolates could be predicted to a high accuracy, our model reported multiple false positives when predicting the success of an introduced isolate, suggesting potential limiting factors not represented in our eco-evolutionary features. Regional models produced for 2 countries reporting the most VpST3 genomes had varying success, reflecting the impacts of class imbalance.

**Conclusions:**

These novel insights into evolutionary features and ecological conditions related to the stages of VpST3 expansion showcase the potential of machine learning models using genomic data and will contribute to the future understanding of the eco-evolutionary pathways of climate-sensitive pathogens.

## Introduction

### Background

Climate change is likely to impact environmentally sensitive pathogens in terms of shifts in seasonality, expansion of suitable habitats, and the emergence and global dispersal of well-adapted variants. This has already been observed for *Vibrio parahaemolyticus* [1], a marine bacterium inhabiting coastal waters that causes acute gastroenteritis when transmitted to humans by ingestion of contaminated seafood, contributing to a large percentage of foodborne infections worldwide. Recent decades have seen this highly adaptable bacterium spread globally and increasingly cause outbreaks [1].

**Figure 1:**
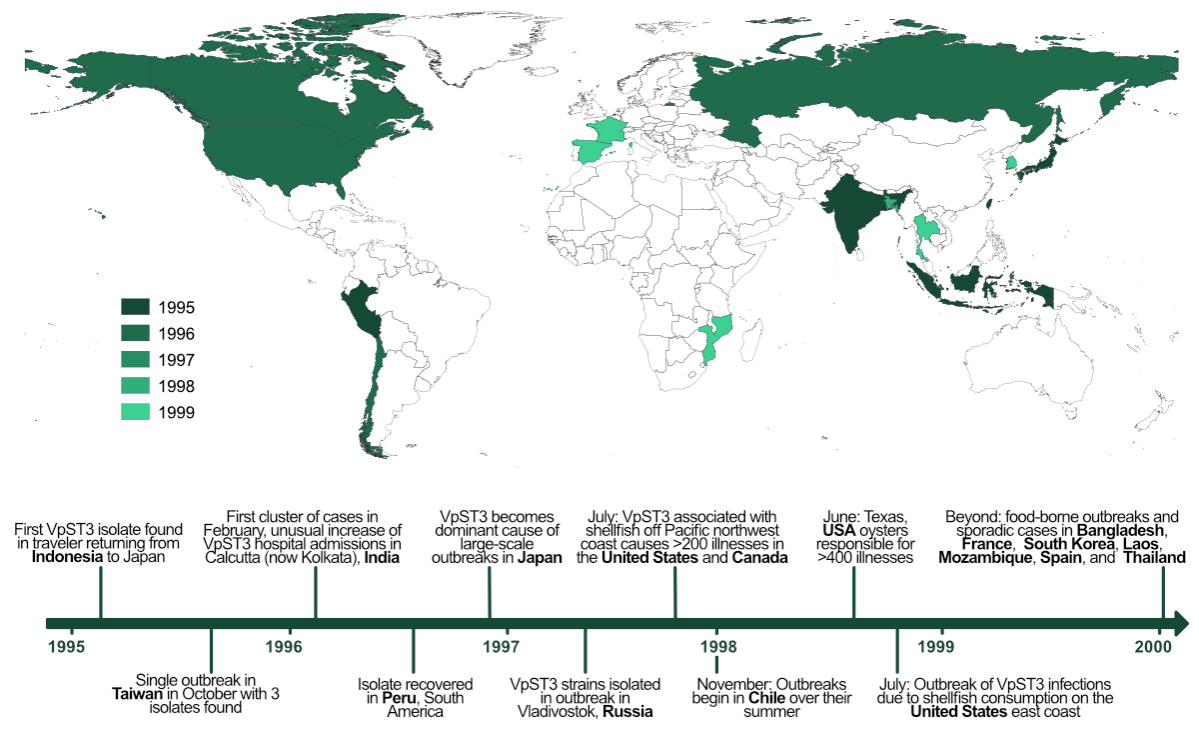
Timeline and map of *Vibrio parahaemolyticus* sequence type 3 (VpST3) initial expansion based on reported isolates and outbreaks in the literature (shapefile provided by Database of Global Administrative Areas).

Before the 1990s, *Vibrio* infections were considered an exotic outcome of travel to Asia, where *Vibrio* bacteria were historically considered endemic. Up to this point, only particular strains of *Vibrio cholerae* had been designated as epidemic variants, characterized by global expansion and pandemic potential. However, transcontinental spread has now been reported for 2 *V parahaemolyticus* clonal types: sequence type 3 from Southeast Asia and, more recently, sequence type 36 from the Pacific Northwest [2,3]. The first of these instances, involving the clonal type *Vibrio parahaemolyticus* sequence type 3 (VpST3), was identified in 1996, when the unique variant, which had not been previously reported, was found to be responsible for up to 80% of the cases in a notable increase of *V parahaemolyticus* infections in Calcutta (now Kolkata), India, in 1996 [4]. This outbreak was unusual, with all recovered isolates clustered into a single homogeneous group, unlike previous outbreaks [3]. Similar isolates were then observed from outbreaks in distinct locations around the world, including Peru, Japan, Russia, Chile, and the United States [5-8] (Figure 1 [4-15]), where the variant was emerging concurrently. This included regions with conditions previously considered adverse for the presence of such pathogens. The epidemic radiations that followed in these diverse regions were the first observed for *V parahaemolyticus* and resulted in the variant supplanting local populations and rapidly becoming the most dominant *V parahaemolyticus* variant globally. As a consequence of this expansion, *V parahaemolyticus* became the second human pathogenic *Vibrio* species with an epidemic nature and, along with *V cholerae*, the only *Vibrio* species with strains capable of worldwide expansions and causing infections at a global level.

This process of expansion results from an epidemic bacterial population structure, as described in the study by Smith et al [16]. Upon a background of numerous rapidly recombining genotypes, a limited number of very frequent genotypes are superimposed, known as clonal complexes, that have originated from highly adaptive ancestral genotypes [16]. The mechanisms behind the rise of these clonal complexes are largely unknown; yet, it is imperative to identify the conditions that allow a pathogen to emerge in such diverse locations and become dominant, as well as the drivers behind these processes, to understand and prepare for future pandemic expansions. When considering environmentally sensitive pathogens such as *Vibrio*, possible drivers can be categorized as either ecological or evolutionary. Evolutionary drivers include the processes of adaptation, mutations that increase fitness, or the uptake or horizontal transfer of beneficial accessory genes. Both ends of the spectrum of genetic diversity—generalists and specialists—are associated with pandemic expansions. Ecological drivers can range from the local environment, which affects pathogen survival and growth, to environmental corridors and transport mechanisms. Importantly, these ecological and evolutionary drivers are not exclusive and, instead, interact significantly, with this interplay known as “eco-evolutionary.” A key example of this would be adaptative selection occurring after arrival to a distinct marine environment. While more studies are considering the coeffect of ecological and evolutionary factors on larger species (such as vertebrates and invertebrates), little attention has been paid to environmentally sensitive pathogens. Focusing on these pathogens would provide novel insights into how particular pathogenic strains emerge [17].

*V parahaemolyticus* is a uniquely placed species, with a history of pandemic expansion that facilitates the study of such eco-evolutionary drivers. First, *Vibrio* are phylogenetically diverse with highly variable genomic backgrounds shaped by recombination and horizontal gene transfer [3], from which specialized variants can emerge. Second, *V parahaemolyticus* exhibits well-characterized environmental thresholds and tolerances, rapidly responding to changes in its marine environment, such as water temperature [18-23] and salinity [18,21,24-26]. Notably, anomalously high temperatures were observed between 1996 and 1998 [27,28] around the emergence of VpST3—pertinent amid the preference of *Vibrio* for warmer waters. From a combined eco-evolutionary perspective, *Vibrio* have high genome plasticity, which facilitates rapid adaptation in response to environmental changes [29], resulting in a large diversity of causative strains and resulting infection dynamics [30,31]. It would be simplistic to assume that all these diverse *V parahaemolyticus* variants respond to environmental change homogeneously, opening up the eco-evolutionary response landscape for exploration.

### Study Objectives

We reconstructed this global expansion using publicly available genome sequences of VpST3 from clinical and environmental sources, isolated from around the world over the period of expansion of this clone, to identify population structure and demographic shifts indicative of the different stages of expansion, including emergence and establishment. We investigated the possible drivers of the expansion and our ability to predict the dynamics of VpST3 by testing a range of evolutionary and ecological drivers in a combined approach using machine learning models to elucidate the complex mechanisms that, when combined, may facilitate such a rapid, global expansion. Machine learning has been credited for its ability to harness the predictive power of evolution, using pattern recognition to uncover complex associations between biological processes [32]; therefore, it is well-placed for the novel exploration of interacting eco-evolutionary mechanisms in combination. Understanding the evolutionary features and ecological conditions related to the stages of pathogen expansion is a crucial step in understanding future eco-evolutionary pathways of climate-sensitive pathogens.

## Methods

The protocol for preprocessing evolutionary and ecological data into a data frame for machine learning analysis is summarized in a graphical representation in Figure S1 in Multimedia Appendix 1.

### Ethical Considerations

Ethical approval was not sought for the present study because it consisted of neither human nor animal experimentation and all genomic datasets used had been previously made publicly available with reference to their ethical approval in the papers associated with these submissions.

### Genomic Data

Raw sequencing datasets from a collection of 311 VpST3 isolates, representing a range of geographic areas, were acquired from public databases for genomic analyses (Table S1 in Multimedia Appendix 1). The 311 isolates covered a temporal range from 1996 to 2021, with 162 (52.1%) from Asia, 78 (25.1%) from North America, and 71 (22.8%) from South America. A series of genetic markers were used to confirm that the isolates were VpST3 using multilocus sequence typing in MLST (version 2.11) [33]. Our analysis was restricted to isolates that were submitted with accompanying isolation date and location details because such metadata were required for the downstream linkage with environmental variables. The raw sequences were processed using default parameters within Bactopia (version 2.0.2) [34], including quality filtering, assembly, and annotation. Core single nucleotide polymorphisms (SNPs) were identified across all sequences using parsnp (version 1.5.6) [35] to create a core genome alignment, mapped to the *V parahaemolyticus* reference genome RIMD2210633. Gubbins (version 3.1.6) [36] was used to remove recombining regions to provide a final nonrecombining core genome alignment.

### Phylogenetic Analysis

TempEst (version 1.5.3) [37] was used to confirm a temporal signal and conformation to a molecular clock, followed by BEAST2 (version 2.7.6) [38] analysis to reconstruct the global phylogenetic dynamics of *V parahaemolyticus*, using BEAUti [39] and a structured coalescent within a MultiTypeTree template [40]. After sensitivity analyses on a range of models, the selected model used a relaxed log normal clock model and a general time reversible (GTR) substitution model, with a normal distribution substitution rate prior. The tip dates and discrete location attributes were used to situate the genomic evolution in space and time. The Markov chain Monte Carlo was run for 250 million states until all outputs converged (effective sample size >200), confirmed by Tracer (version 1.7.1) [41]. The final maximum clade credibility tree was generated using TreeAnnotator within BEAST2.

### Encoding of Expansion Dynamics

The Bayesian phylogenetic analysis and subsequent tree structure informed the designation of a variety of classifications representing VpST3 dynamics. These classifications included populations within the collection, temporal divergence, the success of introductions, and the stages of expansion. We assigned each of these classifications to each of the 311 isolates, using set criteria applied to the phylogenetic tree (Textbox 1), and converted them into binary or categorical features to provide target variables for machine learning analysis.

**Criteria for encoding the numerical and categorical variables of expansion dynamics. The terms in parentheses refer to the column names of the expansion dynamics in the data frame input.**

**Populations (wave)**

Populations within the *Vibrio parahaemolyticus* sequence type 3 (VpST3) collection were identified using TreeStructure (version 0.1.0) [42], which identifies genealogical patterns to infer population structure from time-scaled phylogenies by performing 100,000 tree simulations with a significance threshold set at *P*<.001

**Temporal evolution (earlylate)**

Very few VpST3 isolates were recovered in 2003, after the initial global population expansion; therefore, we specified this year as a split between the early colonizers found before this date and the later isolates recovered after the expansion

**Success (success)**

Failed introductions were monophyletic branches that did not split into further nodes in the phylogenetic tree, while successful introductions were those that saw downstream nodes in the same reported location

**Stages of expansion (stages)**

We split expansion into five defined stages: (1) initial introduction (the first node or nodes in a clade), (2) established population (the nodes in the clade after this introduction), (3) secondary introduction (the first node or nodes in a clade in a new location from the original introduction), (4) secondary establishment (the nodes in the clade after this introduction in the new location), and (5) bottleneck (the last node of a clade or a location within the clade)

**Stages of expansion: binary (stages_binary)**

A simplified version of the previous stages of expansion classification, reducing it to a binary classification of introduced isolates (the first instances in a clade or location) and established isolates (those that followed these introductory nodes within this clade)

### Extraction of Evolutionary Driver Data

Genomic analysis was used to extract features representing possible evolutionary drivers for each isolate. Quantifying the gene content variation in the accessory pangenome in natural populations is important to understand the plasticity and adaptability of populations to environmental perturbations [43]. To obtain a metric of total genes present in each isolate, we used Roary (version 3.13.0) [44] to construct the pangenome and annotate each gene present in each isolate. We used Scoary (version 1.6.16) [45] to identify shell genes (present in 15%-95% of the population) whose presence was statistically associated (*P*<.01) with the previously assigned labels representing introduction, establishment, or success. We retained a selection of these that were common accessory genes (with a presence ranging from 5% to 95% across the isolates in the collection), annotated their function, and generated features representing the binary presence or absence features. We used single-likelihood ancestor counting within HyPhy (version 2.5.48) [46] to estimate the ratio of nonsynonymous to synonymous substitutions (dN/dS) and identify sites under significant diversifying or purifying selection (*P*<.05) in the genes of interest.

SNP mutations of relevance to the expansion process were selected using pcadapt (version 4.3.3) [47] for outlier detection based on population structure. The outliers were inferred based on principal component analysis, using the parameter K=2 and a desired false discovery rate threshold of 0.1 (q-threshold) to identify discriminatory SNP mutations associated with local adaptation. These SNPs were annotated to predict functional effects on genes using SnpEff (version 5.1) [48] and the *V parahaemolyticus* RIMD2210633 genome annotations as a reference. SNPs predicted to have nonsynonymous missense variants were retained for downstream analyses. We recorded the base found at this position for every isolate to assess whether these mutations would help the model define the evolutionary classification. To convert these into numerical values fit for machine learning applications, we reclassified the letters representing bases into numbers (A=1, C=2, T=3, G=4, and N-polymorphic=5).

### Extraction of Ecological Driver Data

Time series data for sea surface temperature (SST) and salinity—2 of the most well-reported environmental drivers of *V parahaemolyticus* in the marine environment [49] —were acquired from the European Centre for Medium-Range Weather Forecasts Reanalysis version 5 [50] and the Met Office Hadley Centre’s EN4.2.2 quality-controlled ocean dataset [51,52], respectively, covering the period from 1995 to 2021. We zonally extracted the climate time series data for the country of isolate recovery, using Database of Global Administrative Areas country zones provided as shapefiles, extending into coastal waters by 2 decimal degrees to extract the local conditions of the marine environment. Although the climate data were available at a monthly resolution, the majority of the genomic isolates only contained an annual resolution. Instead of averaging across the whole year, we created metrics for maximum, minimum, and mean values for each season across the year, alongside generated lagged variables from the previous year. Alongside environmental drivers, the seafood industry, including fisheries [53], seafood consumption and trade [54], and fish market contamination [55], has been previously hypothesized as a possible mechanism for the emergence and spread of *Vibrio* bacteria. We therefore extracted shellfish import data for each country from the FishStatJ database of the Food and Agricultural Organization of the United Nations as annual totals measured in 100 kg of net product weight [56] to explore the potential of this driver.

### Machine Learning Approach

We combined the ecological and evolutionary driver metrics and the classification for each of the criteria into a single data frame for each isolate, with a total of 311 data points.

For our machine learning analysis, we chose a random forest classifier model, an ensemble learning method that uses bootstrapping across decision tree classifiers, due to its high interpretability and implemented the models using the Python module *scikit-learn* (version 1.3.0) [57]. For each expansion dynamic, we created three separate models: 1 model used only the evolutionary drivers as features, a second only ecological drivers as features, and a final model used both ecological and evolutionary drivers in a combined approach. We trained the random forest classifier (using 100 estimators, setting the maximum number of features to consider for best split to the square root of the total number of features, and using bootstrap samples to build trees) on a randomly selected subset of 70.1% (218/311) of the data, retaining the remaining 29.9% (93/311) as an unseen test dataset. In total, there were 109 features used to predict each evolutionary dynamic (Table S2 in Multimedia Appendix 1), of which 60 (55%) represented evolutionary drivers, and 49 (45%) represented ecological drivers. The classification output classes were either binary or categorical based on the expansion dynamic being predicted.

To test the accuracy of these predictions and provide insight into our ability to predict the expansion dynamics of VpST3, we reported 4 accuracy metrics, both per class and across all predictions, when the models were applied to the unseen test data. These metrics included precision (positive prediction rate, affected by false positives), recall (sensitivity rate, affected by false negatives), the *F*_1_-score (a harmonic mean of precision and sensitivity, often used for comparative machine learning performance assessments), and overall accuracy (taking into account all components). We calculated the feature importance for all ecological and evolutionary drivers involved in each model using the Gini importance attribute within the random forest implementation in *scikit-learn* (version 1.3.0) (57), which is computed by the mean and SD of the accumulating impurity decrease within each tree due to the addition of each specific feature.

To assess the collinearity effects from cross-correlations between the ecological and evolutionary driver metrics contained in our model, we calculated the Spearman rank correlation coefficient between the driver variables. During model development of the individual ecological and evolutionary models, we selected features that did not exhibit significant (*P*<.05) collinearity. However, collinearity between evolutionary and ecological features in the combined model was explored, rather than omitted, to gain greater insight into potential eco-evolutionary associations. These significant relationships were visualized in a heat map using the *seaborn* (version 0.12.2) Python library [58].

### Region-Specific Analysis

To explore the potential to understand the successful expansion of VpST3 in particular regions, we developed 2 region-specific models representing an endemic area and an area where VpST3 emerged: China and Peru, respectively. These regions were chosen because they reported the most VpST3 isolates within their respective continents and consist of distinct geographic characteristics to establish whether a regional focus on an area with specific local conditions to drive eco-evolutionary dynamics improves our ability to predict successful expansions. These models were trained and tested on regional subsets of the original data frame, with the same model parameters and features.

## Results

### Phylogeny Characterization

**Figure 2:**
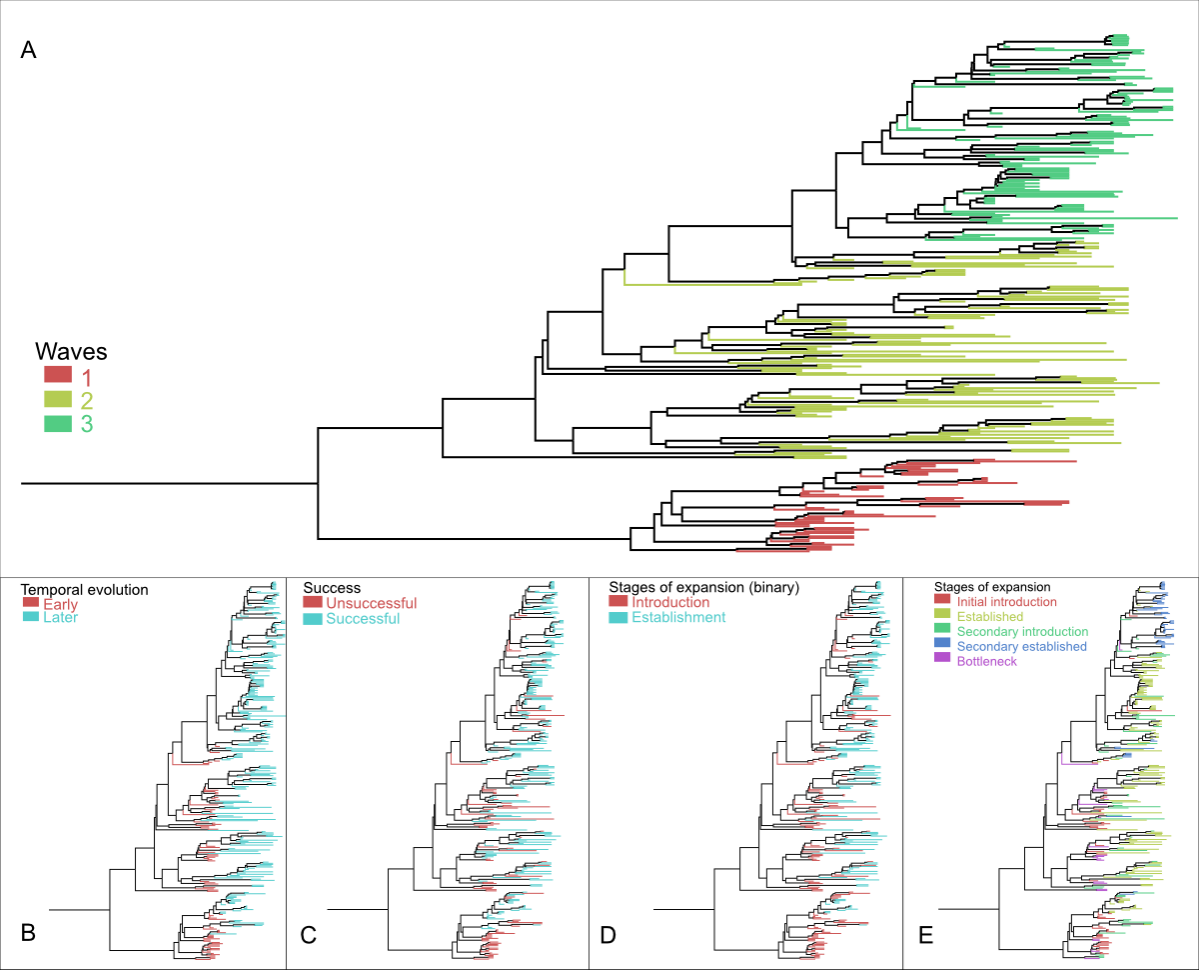
Maximum clade credibility tree with branches color coded by expansion dynamic metrics: (A) population waves, (B) temporal evolution, (C) expansion success, and (D and E) stages of expansion.

The phylogeny revealed an evolutionary population structure within the VpST3 genomes, with multiple introductions into geographically distinct locations, including secondary migrations and introductions. Our phylogenetic analysis found 3 clear population “waves” within VpST3, comprising 56 (18%), 131 (42.1%), and 124 (39.9%) of the 311 isolates (Figure 2A). In terms of temporal evolution, of the 311 isolates, 73 (23.5%) were classified as early colonizers (before 2003), and 238 (76.5%) were isolated after the initial expansion after 2003 (Figure 2B). Regarding expansion success, of the 311 isolates, 86 (27.7%) were classified as unsuccessful and 225 (72.3%) as successful (Figure 2C). With regard to the stages of expansion, of the 311 isolates, under a binary classification, 121 (38.9%) were classified as introduced, with 190 (61.1%) being classified as established (Figure 2D). When this was scaled up to the 5 stages of classification, of the 311 isolates, 49 (15.8%) were classified as initial introductions, 131 (42.1%) as established, 52 (16.7%) as secondary introductions, 58 (18.6%) as secondarily established, and finally 21 (6.8%) as representing bottlenecked populations (Figure 2E).

### Evolutionary Features Extracted

We detected 194 potential adaptive SNP outliers within the collection of genomic isolates, of which 44 (22.7%) were predicted to be missense variants, altering an amino acid within a protein, with predicted moderate effects on particular genes (Table S3 in Multimedia Appendix 1). These SNPs were chosen as evolutionary features for the machine learning analysis. Overall, the total number of genes in each isolate ranged from 4292 to 4735, with no clear temporal signal (*R*^2^=0.08). We identified 400 accessory genes present in 15% to 95% of the entire VpST3 collection and reduced these to 15 (3.8%) genes of interest as evolutionary features for the machine learning analysis (Table 1). This selection was based on genes that were associated with particular expansion metrics; the presence of all 15 selected genes was significantly associated (*P*<.01) with the binary classification delineating introduced and established isolates, and 5 (33%) were further associated with the successful classification metric. Annotation of these genes of interest found that most (n=5, 33%) were functionally associated with survival in the environment and tolerance to environmental conditions (Table 1). In addition, some of them (n=8, 53%) were involved in bacterial transport mechanisms, such as putrescine pathways, that promote biofilm formation. On 4 occasions, 2 versions of a gene with a similar function were identified within this group—for *pilT*, *ttcA*, CARB β-lactamase, and DeoR family transcriptional regulators. Of these 15 accessory genes, no evidence for positive diversifying selection was found (as determined by HyPhy single-likelihood ancestor counting [46]); however, 10 (67%) genes had evidence of negative, purifying selection (*P* value threshold <.10), ranging from 1 to 17 sites under purifying selection.

**Table 1:** Significant associations identified between accessory gene presence and key expansion dynamics.

Annotation	Function	Significance of presence association with classification labels, *P* value		
		Introduction	Establishment	Success
Lactoylglutathione lyase	Enzyme used for methylglyoxal detoxification, contributes to bacterial survival in the environment [59]	<.001	<.001	<.001
HTH^a^-type transcriptional regulator (*puuR*)	Recombinant protein, involved in putrescine pathways [60]	<.001	<.001	<.001
RNA polymerase sigma factor (*RpoS*)	Proteins that regulate transcription in bacteria, activated in response to different environmental conditions	<.001	<.001	—^b^
Type IV pilus twitching motility protein (*pilT*)	Involved in the transport (motility) of the bacteria itself, biofilms, and virulence [61]	<.001	<.001	—
Sodium:proton antiporter	Antiporters (in this case moving sodium ions in or out of a cell) play an important role in tolerance to salt stress [62]	<.001	<.001	<.001
N-carbamoylputrescine amidase (*aguB*)	Involved in biofilm production by converting N-carbamoylputrescine to putrescine [63]	<.001	<.001	<.001
Agmatine deiminase (*aguC*)	Involved in a putrescine pathway [64]	<.001	<.001	<.001
DeoR family transcriptional regulator	Primarily drives the sensing of environmental stimuli and life cycle responses [65]	<.001	<.001	—
Type IV pilus twitching motility protein (*pilT*)	Involved in the transport (motility) of the bacteria itself, biofilms, and virulence [61]	<.001	<.001	—
Carbenicillin-hydrolyzing class A beta-lactamase CARB-23	Expresses β-lactamase for resistance to antibiotic penicillins [66]	<.001	<.001	—
Ribonuclease III (*rnc*)	Modulates pathogenicity: motility, invasiveness, biofilm formation ability, and virulence [67]	<.001	<.001	—
tRNA 2-thiocytidine(32) synthetase (*ttcA*)	Involved in bacterial growth, resistance to biocides, biofilm formation, and swimming motility [68]	<.001	<.001	—
Carbenicillin-hydrolyzing class A beta-lactamase CARB-23	Expresses β-lactamase for resistance to antibiotic penicillins [66]	<.001	<.001	—
DeoR family transcriptional regulator	Primarily drives the sensing of environmental stimuli and life cycle responses [65]	<.001	<.001	—
tRNA 2-thiocytidine(32) synthetase (*ttcA*)	Involved in bacterial growth, resistance to biocides, biofilm formation, and swimming motility [68]	<.001	<.001	—

^a^HTH: helix-turn-helix.

^b^Not applicable.

### Predictive Power

**Figure 3:**
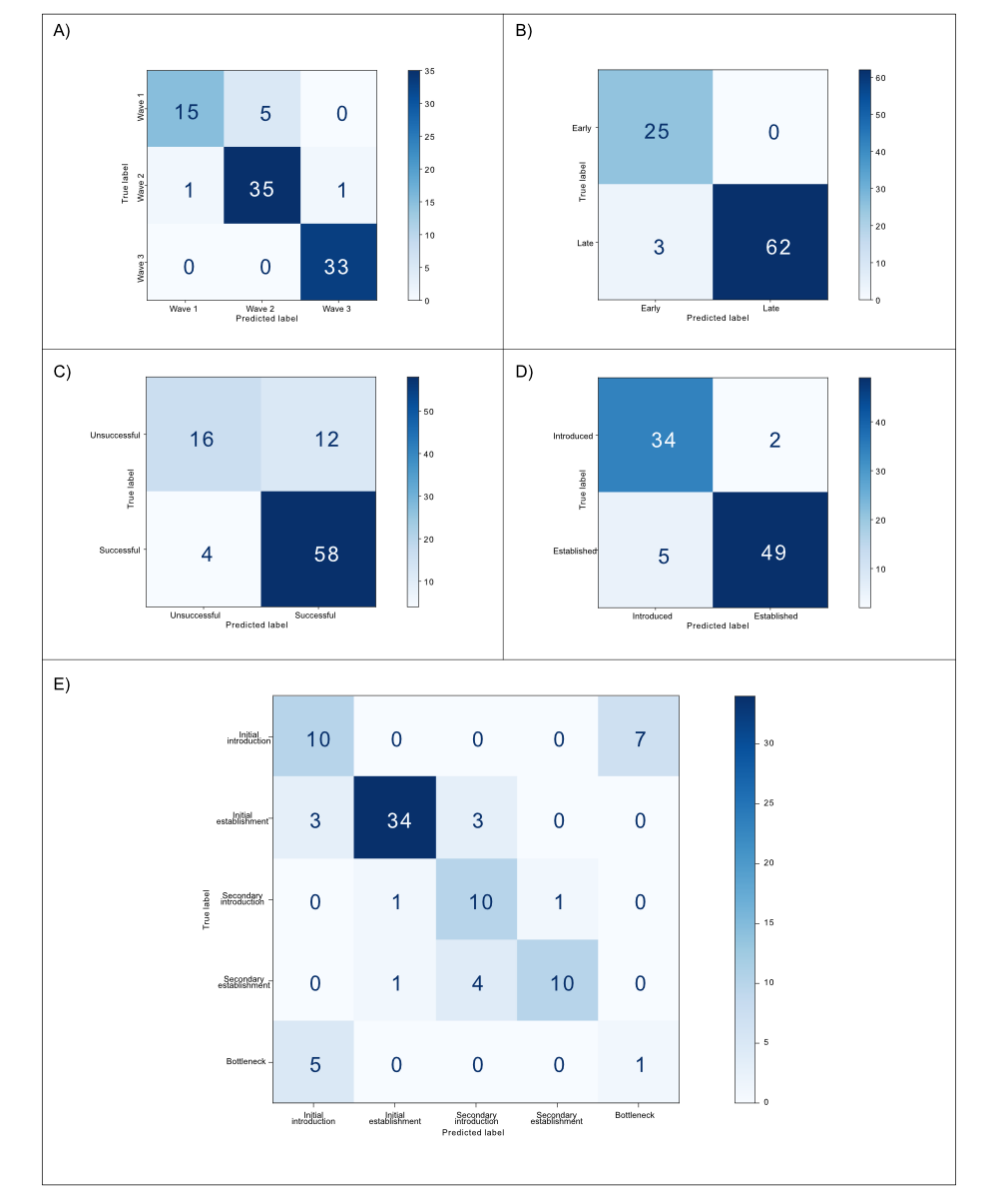
Confusion matrices visualizing the predictions of random forest classifier models for each expansion dynamic when applied to unseen test datasets: (A) population waves, (B) temporal evolution, (C) expansion success, (D) binary stages of expansion, and (E) categorical stages of expansion. (B, C, and D) For binary expansion dynamics, the matrix represents (clockwise from top left) true negatives, false positives, true positives, and false negatives. (A and E) For categorical expansion dynamics, the matrix shows correct class membership and misclassified class memberships for each category.

Overall accuracies for the different expansion metrics ranged from 0.722 to 0.967 for models using a combined eco-evolutionary approach (Figure 3). In our analysis, a combined eco-evolutionary approach almost always improved the accuracy of predicting expansion dynamics compared to using evolutionary or ecological drivers in isolation (Table 2). This was notably apparent for the predictions of the population structure within the phylogeny, in terms of the identification of 3 clear groups, in which evolutionary and ecological features individually produced accuracies of 0.733 and 0.744, respectively, but the combined approach increased the accuracy to 0.922. The only exception occurred when characterizing the success of emergence, where the ecological-only approach achieved the same accuracy as the combined approach. We could distinguish which isolates would be “successfully introduced” to an accuracy of 82% using both ecological and evolutionary data, but 13% of these were false positives, suggesting that our analysis could have overlooked a limiting factor that prevents an isolate from successfully establishing in an area.

**Table 2:** Accuracy metrics of random forest classifier models predicting unseen test data of each expansion dynamic.

Expansion dynamics			Combined eco-evolutionary approach										Evolutionary features only													Ecological features only									
			Precision		Recall		*F*_1_-score		Overall accuracy			Precision			Recall			*F*_1_-score			Overall accuracy			Precision				Recall			*F*_1_-score			Overall accuracy	
**Populations**										0.922												0.733													0.744
	Wave 1	0.938		0.750		0.833					0.529			0.450			0.486						0.900				0.450			0.600					
	Wave 2	0.875		0.946		0.909					0.676			0.676			0.676						0.642				0.919			0.756					
	Wave 3	0.971		1.000		0.985					0.889			0.970			0.928						0.889				0.727			0.800					
	Unweighted average	0.928		0.899		0.909					0.698			0.698			0.697						0.810				0.699			0.719					
	Weighted average	0.924		0.922		0.920					0.721			0.733			0.726			0.733			0.790				0.744			0.737					
**Temporal evolution**										0.967												0.767													0.956
	Early (before 2002)	0.893		1.000		0.943					0.583			0.560			0.571						0.862				1.000			0.926					
	Late (after 2003)	1.000		0.954		0.976					0.833			0.846			0.840						1.000				0.938			0.968					
	Unweighted average	0.946		0.977		0.960					0.708			0.703			0.706						0.931				0.969			0.947					
	Weighted average	0.970		0.967		0.967					0.764			0.767			0.765						0.962				0.956			0.956					
**Stages of expansion**										0.722												0.511													0.733
	Initial introduction	0.556		0.588		0.571					0.412			0.412			0.412						0.526				0.588			0.556					
	Established population	0.944		0.850		0.895					0.605			0.575			0.590						0.944				0.850			0.895					
	Secondary introduction	0.588		0.833		0.690					0.231			0.250			0.240						0.750				0.750			0.750					
	Secondary established population	0.909		0.667		0.769					0.688			0.733			0.710						0.800				0.800			0.800					
	Population bottleneck	0.125		0.167		0.143					0.333			0.333			0.333						0.125				0.167			0.143					
	Unweighted average	0.624		0.621		0.614					0.454			0.461			0.457						0.629				0.631			0.629					
	Weighted average	0.763		0.722		0.735					0.514			0.511			0.512						0.761				0.733			0.745					
**Stages of expansion (binary)**										0.922												0.667													0.911
	Introduction	0.872		0.944		0.907					0.583			0.583			0.583						0.868				0.917			0.892					
	Establishment	0.961		0.907		0.933					0.722			0.722			0.722						0.942				0.907			0.925					
	Unweighted average	0.916		0.926		0.920					0.6533			0.653			0.653						0.905				0.912			0.908					
	Weighted average	0.925		0.922		0.923					0.667			0.667			0.667						0.913				0.911			0.911					
**Success**										0.822												0.711													0.822
	Unsuccessful	0.800		0.571		0.667					0.545			0.429			0.480						0.800				0.571			0.667					
	Successful	0.829		0.935		0.879					0.765			0.839			0.800						0.829				0.935			0.879					
	Unweighted average	0.814		0.753		0.773					0.655			0.634			0.640						0.814				0.753			0.773					
	Weighted average	0.820		0.822		0.813					0.696			0.711			0.700						0.820				0.822			0.813					

Classes that were particularly difficult to predict, with the lowest accuracies reported, were genetic bottlenecks (which were almost always misclassified as initial introductions) and the eco-evolutionary drivers that result in an isolate’s failure to establish successfully. It was harder to predict initial introductions compared to predicting established populations using the categorical “stages of expansion” metric, but when this was reduced to a binary problem, accuracy increased by 0.2, suggesting that separating the stages into initial and secondary introductions (from an established population to a new area) hindered the prediction process.

Exploring the spatiotemporal presence of the errors identified when testing our eco evolutionary models on 90 unseen data points (Table S4 in Multimedia Appendix 1) revealed that the raw highest frequency of errors was found in Asia compared to other continents; however, the relative error rate considering the number of Asian isolates (n=50, 56% of the 90 data points) was the lowest across continents. Notable successes include a strong ability to predict population structure in Asia (in which all 50 samples were accurately predicted) and a low error rate (5.8%) when predicting successful expansions into the United States. Success was more difficult to predict in geographic locations with little representation in the test dataset; or example, there was 1 isolate each from Canada, Japan, Mexico, and Singapore in the test dataset, and only the success of the Canadian isolate was successfully predicted. Temporally, a greater number of errors occurred earlier in the time series, during the initial expansion period of VpST3.

### Eco-Evolutionary Feature Importance

**Figure 4:**
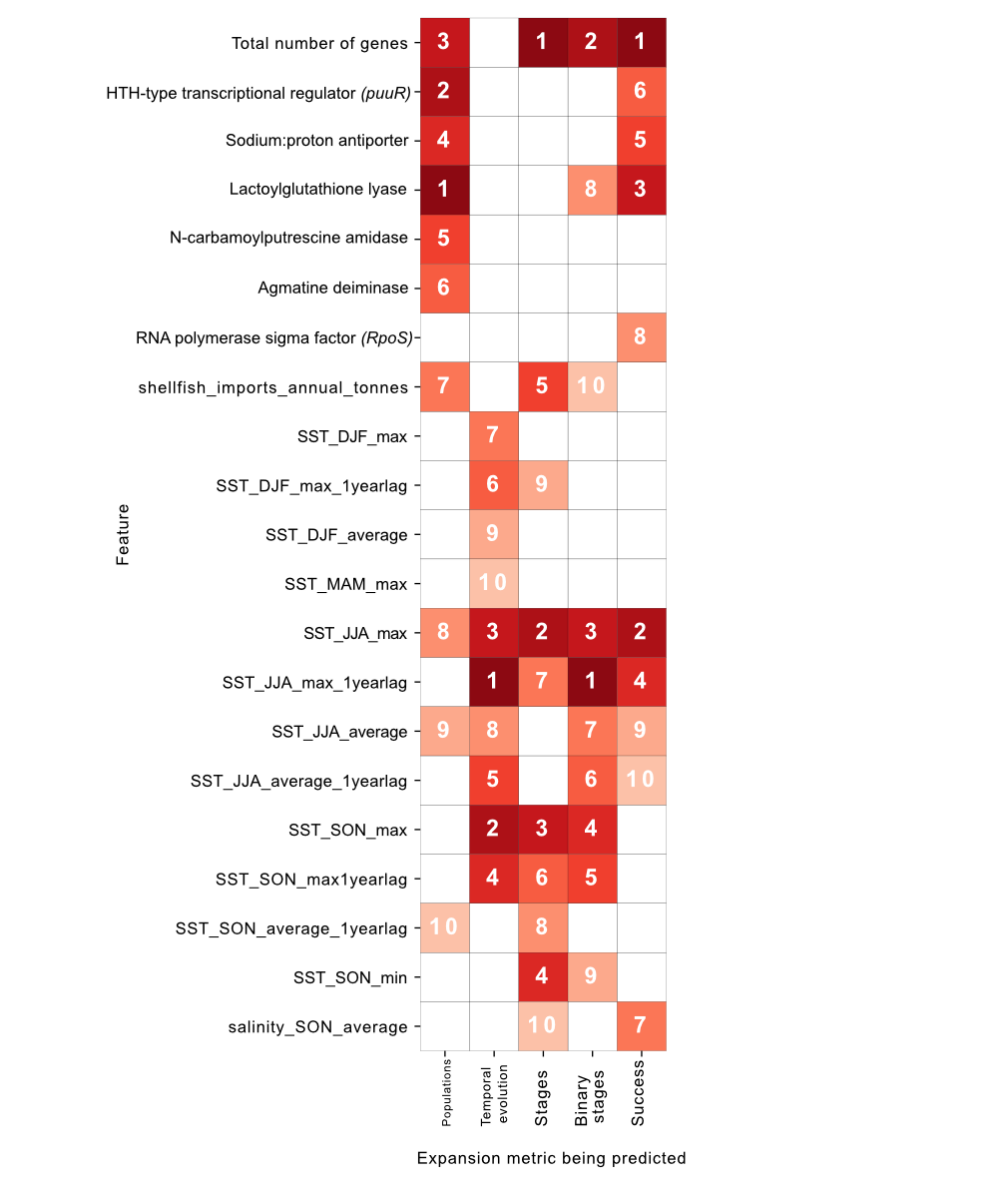
Feature importance for each of the expansion dynamic predictions within each random forest classifier model (where 1 indicates the most important and 10 the 10th most important). DJF: December-January-February; HTH: helix-turn-helix; JJA: June-July-August; MAM: March-April-May; SON: September-October-November; SST: sea surface temperature.

In general, ecological metrics performed stronger than evolutionary metrics individually (Figure S2 in Multimedia Appendix 1). Some of the notably important eco-evolutionary drivers included 3 accessory genes, which were almost always present in introduced isolates (and subsequently eroded) and which provided salt stress tolerance, survival advantages, and biofilm formation for motility, as well as summer maximum sea temperatures from both the year of isolate discovery and the year prior. Of the 109 total features used for training and prediction, the 10 (9.2%) strongest predictive features for each metric, based on feature importance, were collated into a data frame (Figure 4). A small range of these ecological and evolutionary metrics featured within the 10 most important features across all 5 expansion dynamics. For 1 expansion dynamic only—temporal evolution—the strongest predictive features were all environmental features, suggesting that the influence of environmental temporal trends outweighed that of the evolutionary drivers. The total number of genes was an important feature for 4 (80%) of the 5 predicted expansion dynamics and located in the top 3 most important features for each of these, suggesting that genetic diversity was a key distinguishing factor between the classes. In addition, the maximum temperatures during June, July, and August were strong predictor variables, appearing in the top 10 features of all models. Lagged sea temperature effects also offered significant information, notably the SSTs during June, July, and August from the previous year. Although salinity variables did not often appear in the top 10 features, the average salinity during September, October, and November was a useful predictor for classifying the stages of expansion and assessing the success chances of an isolate. Shellfish imports featured as important predictors in classifying population waves and the stages of expansion. Accessory gene presences were stronger predictors in classifying population waves and the chance of success than the stages of expansion themselves.

In terms of notable relationships identified, the success of an isolate was generally associated with higher average and maximum SSTs, particularly during June, July, and August. The presence of certain accessory genes, including *puuR*, *aguB*, and *aguC*, was more important in the classification of “introduced” isolates than in the classification of “established” isolates. The isolates that were predicted to be “introduced” (as opposed to “established”) almost always had these genes present compared to greater variation in the isolates that were predicted to be “established.” This was even more evident in predicting the population to which an isolate belonged, where multiple accessory genes were absent in the third and most recent population wave. Shellfish imports emerged as an important driver in the distinction of the 3 separate populations, with a higher prediction range seen for isolates belonging to the third population wave.

### Cross-Correlation Between Ecological and Evolutionary Drivers

We explored the relationships between the ecological and evolutionary features included in the model and found multiple significant correlations (Figure S3 in Multimedia Appendix 1). Notably, the selected accessory genes exhibited strong correlations with the SST metrics (both positive and negative) as well as with shellfish imports; some genes also correlated with the salinity metrics. In addition, some adaptive SNPs exhibited correlations; for example, the SNP at position 597 had slight negative associations with maximum SSTs and slight positive associations with minimum salinities. The total number of genes had slight positive associations with most of the SST metrics.

### Region-Specific Eco-Evolutionary Models

When generating region-specific models to identify which isolates would be specifically successful in China or Peru, as representative countries, we found the model predictions for China to be largely more accurate, with accuracies ranging from 0.778 to 0.917, compared to model predictions for Peru, with accuracies ranging from 0.529 to 0.706 (Table 3). However, while the model was able to successfully classify successful isolates in China, it had difficulty in classifying the unsuccessful isolates, with poor specificity. The Peru model had more balanced predictions between these 2 classes. In both cases, the ecological features–only model was the best approach, providing the best accuracy. Total gene diversity was the top feature for the combined eco-evolutionary approach (and the evolutionary features–only model). For Peru, the remainder of the top 10 important features were ecological features; however, for China it was an even split between ecological and evolutionary drivers, including the type IV pilus twitching motility protein and the SNP at position 603 (Table S3 in Multimedia Appendix 1), which had not appeared previously among the important features. In the ecological features–only model, the top features were December to February minimum sea temperatures and June to August average temperatures a year prior for China and Peru, respectively.

**Table 3:** Accuracy metrics of region-specific random forest classifier models predicting unseen test data of each expansion dynamic.

Expansion dynamics		Combined eco-evolutionary approach						Evolutionary features only									Ecological features only						
		Precision	Recall	*F*_1_-score	Accuracy		Precision		Recall		*F*_1_-score		Accuracy		Precision			Recall		*F*_1_-score		Accuracy	
**Success in China**						0.889								0.778									0.917
	Unsuccessful	0.500	0.250	0.330			0.167		0.250		0.200				1.000			0.250		0.400			
	Successful	0.912	0.969	0.939			0.900		0.844		0.871				0.914			1.000		0.955			
	Unweighted average	0.706	0.609	0.636			0.533		0.547		0.535				0.957			0.625		0.678			
	Weighted average	0.866	0.889	0.872			0.819		0.778		0.796				0.924			0.917		0.894			
**Success in Peru**						0.529								0.647									0.706
	Unsuccessful	0.500	0.375	0.429			0.750		0.375		0.500				0.714			0.625		0.667			
	Successful	0.545	0.667	0.600			0.615		0.889		0.727				0.700			0.778		0.737			
	Unweighted average	0.523	0.521	0.514			0.683		0.632		0.614				0.707			0.701		0.702			
	Weighted average	0.524	0.529	0.519			0.679		0.647		0.620				0.707			0.706		0.704			

## Discussion

### Principal Findings

Our analysis suggests that VpST3, as a clonal complex, exhibited a high degree of efficacy in propagation during its expansion, evidenced by the numerous introductions in geographically distinct places at similar times. We found evolutionary features that provided mechanisms for this process, including accessory genes linked to functions that facilitate motility and biofilm formation for attachment-based transport mechanisms. The total number of genes within an isolate was an important predictor in the machine learning models for most expansion dynamics. Although we found no trend in gene numbers over time, the model associated higher gene numbers with isolates classified as within established populations, evidenced by a higher prediction range for established isolates. This suggests that isolates that became established could have acquired genes specific to survival in the local conditions, with this plasticity allowing it to colonize new geographic regions. The declining presence of certain accessory genes (*puuR*, *aguB*, and *aguC*) under purifying selection signals suggests that the genes involved in initial introduction may become less useful for population establishment, resulting in reduced selection pressure for these genes. This is corroborated by the prediction ranges of our model for “introduced” isolates, in which these genes were both important features and characterized as almost always present in introduced isolates.

### Assessment of the Eco-Evolutionary Approach

Our analysis has confirmed the hypothesis that considering ecological and evolutionary features in a combined approach to explore the drivers of pathogen expansion yields higher accuracy than dealing with these drivers individually. This is a novel use of the framework described in the study by Campbell et al [17] for characterizing *V parahaemolyticus* expansion dynamics.

From the ecological perspective, SST was a strong predictor variable, as expected from well-established interactions between *V parahaemolyticus* and SST [69]; however, maximum temperatures during June, July, and August emerged as the strongest driver, alongside lagged effects from the previous year. More than two-thirds of our genomic isolates (240/311, 77.2%) were isolated in the northern hemisphere, where these months would be the warmest; this period has previously been described as the “*Vibrio* season” [69]—the characteristics of this season each year seem to drive expansion. In addition, the importance of SSTs in September, October, and November as well as in March, April, and May is pertinent to recent studies that have found expansions in seasonal suitability into cooler months, approximately a 1-month increase every 30 years [70]. Although the period from June to August is the coldest in the southern hemisphere for the South American isolates, it could still drive expansion dynamics when the maximum sea temperatures exceed the minimum for *V parahaemolyticus* survival in the environment, allowing the bacteria to persist in their environmental reservoirs until optimum conditions resume, a phenomenon known as overwintering [71]. Sea temperatures can drive both survival and community composition changes [22,72], with mostly positive associations between SST and the successful established isolates in our analyses. In laboratory studies, increases in seawater temperature have been found to upregulate the expression of virulence factors involved in adhesion processes, such as biofilm formation [73], which could facilitate transport mechanisms via attachment to marine organisms that aid expansion and settlement in new areas.

Generally, the models using evolutionary features only had a lower predictive potential; however, the inclusion of evolutionary features improved the ecological models when combined. The evolutionary features themselves potentially did not offer enough predictive information independently, but when linked to the specific local environmental conditions in which the evolutionary processes provide survival benefits, the evolutionary features were able to provide useful information within the model on pathogen expansion. The evolutionary features might lack meaning outside of ecological contexts or indeed play a different biological role in different ecological contexts. This is supported by the cross-correlations identified between several evolutionary features and the associated environmental conditions (Figure S3 in Multimedia Appendix 1), indicating that these interacting factors themselves, in the form of dynamic evolutionary responses to environmental conditions, can provide predictors of pathogen expansion. This justifies the inclusion of both ecological and evolutionary features in the same predictive model to account for the interactions between them. We observed a specific eco-evolutionary mechanism in our analysis, where SSTs were significantly associated with the presence of multiple accessory genes (Figure S3 in Multimedia Appendix 1), which could indicate an introduced selection pressure in the environment, with changes in SST representing a myriad of implications for the microbial community. However, it is important to note that these cross-correlations provide limited information and could also be purely reflecting the strength of the temporal trends of accessory gene presence, as the result of 2 concurrent or diverging trends, with sea temperature gradually increasing over the time period and accessory gene presence either increasing or decreasing steadily.

Shellfish imports were an important driver for the classification of the third population wave, which could allude to a population opportunistically taking advantage of shellfish movements as a transport mechanism. This would explain why this population has purged multiple accessory genes offering transport mechanisms, such as biofilm pathways. While the role of live aquatic animal transport in contributing to *V parahaemolyticus* expansion is currently unclear, studies have found that this method of transport introduces new populations, facilitates the exchange of genetic material, and promotes adaptation [74]. Further analysis will need to explore whether this subpopulation has undergone innovation to improve host-pathogen attachment mechanisms, particularly involving shellfish.

Few of the SNP mutations identified during outlier detection featured heavily in model decisions, despite our methodology aiming to identify mutations affecting proteins that could promote expansion dynamics. While we encoded the SNPs as categorical features in our machine learning analysis, alternative encoding techniques, such as one-hot encoding, have been explored, and it was found that including information on not only the mutation but also the position of mutation can improve accuracy [75]. Further analysis or different approaches should be explored to improve the identification of mutations critical to expansion processes.

While the models were designed generically to predict a range of expansion metrics, they could be further refined for specific purposes. There were several instances of a large discrepancy between recall and precision, particularly for smaller, underrepresented classes such as bottlenecks, which is a common issue in machine learning when dealing with imbalanced datasets. The models here were not developed individually to obtain the greatest accuracy, as the aim was to facilitate the comparison of accuracy metrics when combining ecological and evolutionary features. However, these imbalances can be remedied on a per-model basis in the future using techniques such as class weights to assign higher weights to minority classes during training or through oversampling (of the minority classes) and undersampling (of the majority classes), as demonstrated by DeLuca et al [76]. The difficulties in separating initial introductions and bottlenecks can be simplified into understanding why a particular introduction is successful or unsuccessful. We did find a potential limiting factor when predicting this success as a separate expansion metric, resulting in a high proportion of false positives where unsuccessful isolates were misclassified as successful.

We propose that a potential limiting factor here could be plankton presence, which has been found to offer nutrients for growth and host protection [77], which was not included in the analysis. This is relevant given the biofilm-related accessory genes identified, which facilitate attachment to plankton, in which these eco-evolutionary factors could combine to provide further information on isolate success. Similarly, plankton abundance was found to significantly increase the presence of 2 major virulence factors of *V parahaemolyticus*, *tdh* and *trh* [78], underlining another eco-evolutionary mechanism driving *V parahaemolyticus* dynamics. There are difficulties in quantifying marine plankton presence for such a global collection spanning decades. Earth Observation data offers a suitable source for ecological driver data in the future, providing consistent time series data at a sufficient resolution; however, satellite observations of plankton (using chlorophyll-a concentration as a proxy) are only available from late 1997; the key preceding year that represents the pivotal early introductions of the expansion of VpST3 is missing.

The spatiotemporal trends of error counts discussed (Table S4 in Multimedia Appendix 1) offer insights into model limitations and areas for future improvement, such as improving our predictive capabilities during the initial emergence of a pathogen strain and in geographic regions reporting few isolates (as is common during initial expansion).

### Regional Predictive Performance for a Globally Expanding Pathogen

The difference in accuracy between the China and Peru regional models is likely due to the consequences of class imbalances. The Chinese isolates had a much higher proportion of successful isolates (108/120, 90%) than Peru (27/55, 49%), which meant that, although we were able to predict successful isolates with high precision and recall, it was very difficult to predict the minority class of unsuccessful isolates (*F*_1_-score=0.33). Such class imbalances result in overfitting of the majority class, enabling the model to achieve a high accuracy of 90% even if it simply predicted all isolates to be successful. This can be seen in the China model using only ecological features, in which the majority class (successful isolates) was predicted perfectly due to 97% (35/36) of the data points being predicted as successful. Further evidence for overfitting is provided by a large difference between the area under the receiver operating characteristic curve values of the training and test data, which were 0.860 and 0.949, respectively. To overcome such overfitting during the future development of regional models, per-class and alternative accuracy metrics need to be considered and imbalances addressed through methods previously outlined. In the Peru model, the number of successful and unsuccessful isolates were much more balanced, resulting in lower but more balanced per-class accuracy metrics. Currently, this would suggest that we can predict the success of a pathogenic variant isolate more accurately in an endemic region than in an emerging one but at the expense of possible overfitting, providing areas for improvement. In both cases, we found that ecological drivers alone were the best approach, suggesting that the evolutionary features were introducing noise into the model. This suggests that focusing on common features in the whole group that might facilitate expansion on a global scale might not be as valuable as more region-specific evolutionary drivers, such as those representative of adaptation to local conditions of a particular region, which would need to be extracted for a more successful regional approach.

However, it is important to note that while models can be improved specifically for particular geographic regions, for example, based on the ranges of local environmental conditions, this comes at the expense of declining applicability. Such applicability could be seen as a priority for a globally expanding pathogen such as VpST3, requiring a model that is able to function in a range of distinct geographic regions. Future work could mediate this trade-off through the introduction of regional encoders as features [24] or through engineering environmental features to be more comparable, such as through normalized anomalies rather than raw values.

### Future Predictive Potential

While this analysis focused on the 3 continents reporting the most VpST3 isolates (Asia, North America, and South America), in the future, the focus will need to shift to countries that lie on the periphery of the environmental tolerance ranges of *V parahaemolyticus*, representing the potential locations of future expansion. These include Europe, which, in recent years, has observed the emergence of *Vibrio* lineages and increases in vibriosis incidence as an emerging public health issue [79]. Increased genomic surveillance is required in these countries to test the ability of this framework to identify expansion potential into these new regions.

In addition, the eco-evolutionary analysis was limited by the annual resolution of the genomic isolate metadata and shellfish movement data. The majority of the isolates in our collection were submitted to public databases with limited metadata, specifying only a country and a year; however, higher-resolution metadata, such as a district and a day, week, or month, as suggested by Campbell et al [17], would greatly improve the specificity of the related ecological data that we could then append to this isolate, which is available at a very high resolution. This is particularly necessary to account for the rapid evolutionary timescales on which bacteria such as *Vibrio* function [29]. Future models would benefit from higher spatiotemporal–resolution datasets for machine learning training that facilitate the characterization of more specific eco-evolutionary drivers and increase predictive accuracy.

### Conclusions

This pilot study provides a precedent for combining ecological and evolutionary driver data using machine learning to predict pathogen expansion metrics. This both aids our understanding of historic expansion and, through further refinement and development, could be operationalized into a trained database through which a new recovered isolate could be submitted and predictions made as to its introduction or establishment potential to track pathogen expansion in near real time. The current limitations preventing such operationalization include sufficient genomic surveillance, data accessibility, and interdisciplinary analysis requirements. Accuracy would need to be refined to the appropriate confidence values based on user requirements of model sensitivity. Further exploration of applicability to a range of climate-sensitive pathogens will require sufficient genomic surveillance, which is currently limited by poor spatiotemporal resolution. Combining state-of-the art analyses of both ecological and evolutionary pathogen drivers will provide new insights into future eco-evolutionary pathways of climate-sensitive pathogens.

## Appendix

Multimedia Appendix 1 Supplementary tables and figures.

## Abbreviations

dN/dS: ratio of nonsynonymous to synonymous substitutions
GTR: general time reversible
SNP: single nucleotide polymorphism
SST: sea surface temperature
VpST3: Vibrio parahaemolyticus sequence type 3
