# Supplementary material for: Eco-Evolutionary Drivers of Vibrio parahaemolyticus Sequence Type 3 Expansion: Retrospective Machine Learning Approach
Source: JMIR Bioinform Biotechnol. 2024 Nov 28;5:e62747. doi: 10.2196/62747 (PMC11638695; doi:10.2196/62747)
Supplement: Multimedia Appendix 1 [file bioinform_v5i1e62747_app1.docx]

**Supplementary Information**

Supplementary Table 1- Accession numbers and metadata for VpST3 genomes used within this study

| **Strain** | **Collection date** | **Country** | **Region** | **BioIsolate** | **BioProject** |
| --- | --- | --- | --- | --- | --- |
| 605 | 2006 | USA |  | SAMN02597368 | PRJNA176634 |
| 658 | 2007 | USA | Washington | SAMN05195070 | PRJNA324096 |
| 752 | 2007 | USA | Washington | SAMN05194683 | PRJNA324080 |
| 765 | 2007 | USA | Washington | SAMN05195120 | PRJNA324105 |
| 783 | 2007 | USA | Washington | SAMN05195061 | PRJNA324095 |
| 863 | 2007 | USA | Washington | SAMN02204307 | PRJNA188204 |
| 920 | 2007 | USA | Washington | SAMN05194682 | PRJNA324079 |
| 4635 | 2007 | USA | Washington | SAMN05220835 | PRJNA325137 |
| 4703 | 2007 | USA | Washington | SAMN05220837 | PRJNA325139 |
| 100138 | 08/05/2008 | China | Guangdong | SAMN05935524 | PRJNA350230 |
| 100145 | 08/06/2013 | China | Guangdong | SAMN05935529 | PRJNA350230 |
| 100150 | 12/10/2008 | China | Guangdong | SAMN05935534 | PRJNA350230 |
| 100151 | 28/06/2010 | China | Guangdong | SAMN05935535 | PRJNA350230 |
| 100152 | 12/07/2010 | China | Guangdong | SAMN05935536 | PRJNA350230 |
| 100153 | 19/05/2009 | China | Guangdong | SAMN05935537 | PRJNA350230 |
| 210265 | 14/05/2018 | USA | NewJersey | SAMN18528236 | PRJNA706389 |
| 237135 | 2004 | Canada | Ontario | SAMN03349598 | PRJNA275536 |
| 237865 | 2004 | Canada | Ontario | SAMN03452289 | PRJNA275536 |
| 511793 | 2017 | USVirginIslands |  | SAMN09280063 | PRJNA438219 |
| 524167 | 2017 | Thailand |  | SAMN09280067 | PRJNA438219 |
| 533448 | 2018 | Malaysia |  | SAMN09280070 | PRJNA438219 |
| 004-02 | 2002 | Peru | Lima | SAMN40891098 | PRJNA1062747 |
| 020-02 | 2002 | Peru | Lima | SAMN40891099 | PRJNA1062747 |
| 056-01 | 2001 | Peru | Lambayeque | SAMN40891102 | PRJNA1062747 |
| 085-02 | 2002 | Peru | Lima | SAMN40891103 | PRJNA1062747 |
| 091-10 | 2010 | Peru |  | SAMN15428964 | PRJNA643807 |
| 092-10 | 2010 | Peru |  | SAMN15428968 | PRJNA643807 |
| 1171-97 | 1997 | Peru | Moquegua | SAMN40891089 | PRJNA1062747 |
| 1218-11 | 2011 | Peru |  | SAMN15428974 | PRJNA643807 |
| 164-16 | 2016 | Peru |  | SAMN12364846 | PRJNA556706 |
| 165-16 | 2016 | Peru |  | SAMN15429002 | PRJNA643807 |
| 182-02 | 2002 | Peru | Chiclayo | SAMN40891104 | PRJNA1062747 |
| 2214-17 | 2017 | Peru |  | SAMN12364848 | PRJNA556706 |
| 240-02 | 2002 | Peru | Lima | SAMN40891105 | PRJNA1062747 |
| 2434-00 | 2000 | Peru |  | SAMN15428944 | PRJNA643807 |
| 249-15 | 2015 | Peru |  | SAMN12364841 | PRJNA556706 |
| 2568-00 | 2000 | Peru |  | SAMN15428945 | PRJNA643807 |
| 276-99 | 1999 | Peru | Lima | SAMN40891093 | PRJNA1062747 |
| 277-15 | 2015 | Peru |  | SAMN15428982 | PRJNA643807 |
| 278-99 | 1999 | Peru | Lima | SAMN40891094 | PRJNA1062747 |
| 293-10 | 2010 | Peru |  | SAMN15428966 | PRJNA643807 |
| 325-00 | 2000 | Peru |  | SAMN15428940 | PRJNA643807 |
| 327-00 | 2000 | Peru |  | SAMN15428942 | PRJNA643807 |
| 361-10 | 2010 | Peru |  | SAMN15428969 | PRJNA643807 |
| 403-00 | 2000 | Peru |  | SAMN15428941 | PRJNA643807 |
| 404-00 | 2000 | Peru |  | SAMN15428938 | PRJNA643807 |
| 405-00 | 2000 | Peru | Lambayeque | SAMN40891095 | PRJNA1062747 |
| 429-00 | 2000 | Peru | Lima | SAMN40891096 | PRJNA1062747 |
| 454-10 | 2010 | Peru |  | SAMN15428970 | PRJNA643807 |
| 461-00 | 2000 | Peru | Lima | SAMN40891090 | PRJNA1062747 |
| 462-00 | 2000 | Peru | Lima | SAMN40891091 | PRJNA1062747 |
| 568-00 | 2000 | Peru | Huaral | SAMN40891100 | PRJNA1062747 |
| 572-01 | 2001 | Peru | Iquitos | SAMN40891097 | PRJNA1062747 |
| 686-17 | 2017 | Peru |  | SAMN12364847 | PRJNA556706 |
| 706-00 | 2000 | Peru | Lima | SAMN40891101 | PRJNA1062747 |
| 706-00 | 2000 | Peru |  | SAMN15428939 | PRJNA643807 |
| 763-97 | 1997 | Peru | Chancay | SAMN40891084 | PRJNA1062747 |
| 784-98 | 1998 | Peru | Lima | SAMN40891082 | PRJNA1062747 |
| 790-97 | 1997 | Peru | Cajamarca | SAMN40891085 | PRJNA1062747 |
| 859-07 | 2007 | Peru |  | SAMN40891108 | PRJNA1062747 |
| 875-97 | 1997 | Peru | Lambayeque | SAMN40891086 | PRJNA1062747 |
| 906-97 | 1997 | Peru | Lima | SAMN40891087 | PRJNA1062747 |
| 948-97 | 1997 | Peru | Lambayeque | SAMN40891088 | PRJNA1062747 |
| 971-98 | 1998 | Peru | Lima | SAMN40891083 | PRJNA1062747 |
| 974-98 | 1998 | Peru | Lima | SAMN40891112 | PRJNA1062747 |
| A10_780-98 | 1998 | Peru |  | SAMN40891081 | PRJNA1062747 |
| A4EZ927 | 2004 | Canada | BritishColumbia | SAMN04327443 | PRJNA304021 |
| A5Z853 | 2005 | Canada | BritishColumbia | SAMN04377388 | PRJNA304021 |
| AN-5034 | 1998 | Bangladesh |  | SAMN02436265 | PRJNA33625 |
| AQ4901 | 2000 | Peru | Lima | SAMN40891092 | PRJNA1062747 |
| ATC210 | 1998 | Chile | Antofagasta | SAMN40891111 | PRJNA1062747 |
| ATC220 | 1998 | Chile | Antofagasta | SAMN02781334 | PRJNA233509 |
| ATC224 | 1998 | Chile | Antofagasta | SAMN40891107 | PRJNA1062747 |
| BH0083 | 2020-08 | China | Beihai | SAMN20294544 | PRJNA747744 |
| CAIM 1400 | 2004 | Mexico |  | SAMN13893117 | PRJNA602337 |
| CDC_K4637W | 01/10/2006 | USA | NewYork | SAMN03358829 | PRJNA273159 |
| CDC_K4775 | 24/02/2007 | USA | Georgia | SAMN02368289 | PRJNA273159 |
| CDC_K5010G | 16/09/2006 | USA | Massachusetts | SAMN02368298 | PRJNA273159 |
| CDC_K5010W | 16/09/2006 | USA | Massachusetts | SAMN02368299 | PRJNA273159 |
| CDC_K5058 | 15/05/2007 | USA | Texas | SAMN02368300 | PRJNA273159 |
| CDC_K5528 | 06/10/2007 | USA | Georgia | SAMN02368334 | PRJNA273159 |
| CEFAS0002 | 2009 | Vietnam |  | SAMN12254028 | PRJNA438219 |
| CEFAS0003 | 2009 | Thailand |  | SAMN12254029 | PRJNA438219 |
| CEFAS0004 | 2013 | Thailand |  | SAMN12254030 | PRJNA438219 |
| CEFAS0006 | 2010 | Thailand |  | SAMN12254032 | PRJNA438219 |
| CEFAS0008 | 2014 | Cuba |  | SAMN12254034 | PRJNA438219 |
| CEFAS0010 | 2014 | Thailand |  | SAMN12254036 | PRJNA438219 |
| CEFAS0011 | 2014 | Thailand |  | SAMN12254037 | PRJNA438219 |
| CEFAS0015 | 2011 | Thailand |  | SAMN12254041 | PRJNA438219 |
| CEFAS0017 | 2016 | Thailand |  | SAMN12254043 | PRJNA438219 |
| CEFAS0023 | 2016 | Colombia |  | SAMN12254049 | PRJNA438219 |
| CFSAN007450 | 22/08/2012 | USA | Maryland | SAMN02741386 | PRJNA245882 |
| CFSAN007451 | 24/08/2012 | USA | Maryland | SAMN02741387 | PRJNA245882 |
| CFSAN018757 | 1997 | Peru |  | SAMN03941065 | PRJNA245882 |
| CFSAN023533 | 1996 | India |  | SAMN06076986 | PRJNA245882 |
| CFSAN023535 | 1996 | India |  | SAMN06076987 | PRJNA245882 |
| CFSAN023536 | 1996 | India |  | SAMN06076988 | PRJNA245882 |
| CFSAN023537 | 1997 | India |  | SAMN06076989 | PRJNA245882 |
| CFSAN023538 | 1998 | Bangladesh |  | SAMN06076990 | PRJNA245882 |
| CFSAN023540 | 1998 | SouthKorea |  | SAMN06076991 | PRJNA245882 |
| CFSAN023541 | 1998 | USA | Texas | SAMN06076992 | PRJNA245882 |
| CFSAN023542 | 1998 | USA | NewYork | SAMN06076993 | PRJNA245882 |
| CFSAN023543 | 1998 | USA | NewYork | SAMN06076994 | PRJNA245882 |
| CFSAN023544 | 1998 | USA | NewYork | SAMN06076995 | PRJNA245882 |
| CFSAN023545 | 1998 | Bangladesh |  | SAMN06076996 | PRJNA245882 |
| CFSAN023546 | 1999 | Bangladesh |  | SAMN06076997 | PRJNA245882 |
| CFSAN023547 | 1999 | Thailand |  | SAMN06076998 | PRJNA245882 |
| CFSAN023548 | 1998 | Bangladesh |  | SAMN06076999 | PRJNA245882 |
| CFSAN023556 | 2005 | Chile |  | SAMN06077007 | PRJNA245882 |
| CFSAN023563 | 1999 | Thailand |  | SAMN06077014 | PRJNA245882 |
| CFSAN023564 | 1998 | Japan |  | SAMN06077015 | PRJNA245882 |
| CFSAN023566 | 2000 | Bangladesh |  | SAMN06077017 | PRJNA245882 |
| CFSAN026732 | 2014 | USA |  | SAMN08370015 | PRJNA245882 |
| CFSAN029652 | 2009 | Peru |  | SAMN08225458 | PRJNA245882 |
| CFSAN029655 | 2009 | Peru |  | SAMN08225457 | PRJNA245882 |
| CFSAN029657 | 2009 | Peru |  | SAMN08225459 | PRJNA245882 |
| CICESE-186 | 1999 | Mexico |  | SAMN13893119 | PRJNA602337 |
| DH13 | 2018 | China | Dalian | SAMN15889525 | PRJNA633360 |
| EKP-026 | 2008 | Bangladesh |  | SAMN02641511 | PRJNA176652 |
| F2_10 | 2014 | China | Guangxi | SAMN07338135 | PRJNA393608 |
| F2_7 | 2014 | China | Guangxi | SAMN07338141 | PRJNA393608 |
| F2_8 | 2014 | China | Guangxi | SAMN07338142 | PRJNA393608 |
| F2_9 | 2014 | China | Guangxi | SAMN07338143 | PRJNA393608 |
| F3_1 | 2014 | China | Guangxi | SAMN07338144 | PRJNA393608 |
| F3_10 | 2014 | China | Guangxi | SAMN07338145 | PRJNA393608 |
| F3_7 | 2014 | China | Guangxi | SAMN07338151 | PRJNA393608 |
| F3_8 | 2014 | China | Guangxi | SAMN07338152 | PRJNA393608 |
| F3_9 | 2014 | China | Guangxi | SAMN07338153 | PRJNA393608 |
| F63267 | 2006 | Canada | BritishColumbia | SAMN04422055 | PRJNA304021 |
| FDAARGOS_191 | 1996 | India |  | SAMN04875528 | PRJNA231221 |
| G1 | 2014 | Peru |  | SAMN12364839 | PRJNA556706 |
| G1_3 | 2014 | China | Guangxi | SAMN07338213 | PRJNA393608 |
| G1_8 | 2014 | China | Guangxi | SAMN07338218 | PRJNA393608 |
| G1_9 | 2014 | China | Guangxi | SAMN07338219 | PRJNA393608 |
| G6 | 2015 | Peru | Lima | SAMN15428983 | PRJNA643807 |
| G8 | 2016 | Peru | Lima | SAMN15429003 | PRJNA643807 |
| GIMxtfL61-2011.05 | 2011-05 | China | Shenzhen | SAMN06162268 | PRJNA357947 |
| GIMxtfL65-2011.05 | 2011-05 | China | Shenzhen | SAMN06163187 | PRJNA357986 |
| Gxw_7004 | 09/06/2007 | China | Guangxi | SAMN04349747 | PRJNA306401 |
| H11 | 2015 | Peru | Lima | SAMN15428984 | PRJNA643807 |
| H12 | 2016 | Peru | Lima | SAMN15429004 | PRJNA643807 |
| ICDC-VP01784 | 2016 | China | Zhenjiang | SAMN15294466 | PRJNA639932 |
| ICDC-VP01786 | 2016 | China | Suqian | SAMN15294467 | PRJNA639932 |
| ICDC-VP01787 | 2016 | China | Wuxi | SAMN15294468 | PRJNA639932 |
| ICDC-VP01791 | 2016 | China | Nantong | SAMN15294469 | PRJNA639932 |
| ICDC-VP01794 | 2017 | China | Huaian | SAMN15294471 | PRJNA639932 |
| ICDC-VP01797 | 2017 | China | Nanjing | SAMN15294472 | PRJNA639932 |
| ICDC-VP01799 | 2017 | China | Nanjing | SAMN15294473 | PRJNA639932 |
| ICDC-VP01800 | 2017 | China | Nanjing | SAMN15294474 | PRJNA639932 |
| ICDC-VP01801 | 2017 | China | Nantong | SAMN15294475 | PRJNA639932 |
| ICDC-VP01802 | 2017 | China | Nantong | SAMN15294476 | PRJNA639932 |
| ICDC-VP01803 | 2017 | China | Wuxi | SAMN15294477 | PRJNA639932 |
| ICDC-VP01805 | 2018 | China | Nanjing | SAMN15294478 | PRJNA639932 |
| ICDC-VP01812 | 2018 | China | Wuxi | SAMN15294482 | PRJNA639932 |
| ICDC-VP01813 | 2018 | China | Suqian | SAMN15294483 | PRJNA639932 |
| ICDC-VP01814 | 2018 | China | Yangzhou | SAMN15294484 | PRJNA639932 |
| ICDC-VP01815 | 2018 | China | Yangzhou | SAMN15294485 | PRJNA639932 |
| ICDC-VP01816 | 2018 | China | Suqian | SAMN15294486 | PRJNA639932 |
| L1 | 2015 | China | Guangzhou | SAMN14411226 | PRJNA613630 |
| L3 | 2015 | China | Guangzhou | SAMN14411228 | PRJNA613630 |
| L7 | 2015 | China | Guangzhou | SAMN14411231 | PRJNA613630 |
| L70 | 16/04/2016 | China |  | SAMN05890675 | PRJNA347505 |
| L8 | 2015 | China | Guangzhou | SAMN14411232 | PRJNA613630 |
| PMA37.5 | 2005-01 | Chile |  | SAMN05858273 | PRJNA345099 |
| PMC16.7 | 2007 | Chile | Puerto Montt | SAMN40891106 | PRJNA1062747 |
| PMC39.5 | 2005 | Chile | Puerto Montt | SAMN40891110 | PRJNA1062747 |
| PMC48 | 2004 | Chile | PuertoMontt | SAMN02781337 | PRJNA233509 |
| PMC73.7 | 2007 | Chile | Puerto Montt | SAMN40891109 | PRJNA1062747 |
| PNUSAV000055 | 29/04/2017 | USA | California | SAMN07559594 | PRJNA266293 |
| PNUSAV000060 | 15/05/2017 | USA | Massachusetts | SAMN07327345 | PRJNA266293 |
| PNUSAV000061 | 09/06/2017 | USA | Massachusetts | SAMN07327346 | PRJNA266293 |
| PNUSAV000101 | 22/08/2017 | USA | Maryland | SAMN07838949 | PRJNA266293 |
| PNUSAV000140 | 08/09/2017 | USA | Pennsylvania | SAMN08113868 | PRJNA266293 |
| PNUSAV000173 | 22/01/2018 | USA | California | SAMN08627210 | PRJNA266293 |
| PNUSAV000174 | 03/02/2018 | USA | Alaska | SAMN08627207 | PRJNA266293 |
| PNUSAV000186 | 18/04/2018 | USA | Pennsylvania | SAMN09689475 | PRJNA266293 |
| PNUSAV000189 | 21/05/2018 | USA | Maryland | SAMN09488306 | PRJNA266293 |
| PNUSAV000202 | 05/07/2018 | USA | Maryland | SAMN09671577 | PRJNA266293 |
| PNUSAV000207 | 02/07/2018 | USA | Maryland | SAMN09671587 | PRJNA266293 |
| PNUSAV000211 | 06/07/2018 | USA | Virginia | SAMN09726939 | PRJNA266293 |
| PNUSAV000223 | 02/07/2018 | USA | Maryland | SAMN09703857 | PRJNA266293 |
| PNUSAV000253 | 18/06/2018 | USA | SouthCarolina | SAMN12212336 | PRJNA266293 |
| PNUSAV000255 | 14/07/2018 | USA | Oregon | SAMN09745133 | PRJNA266293 |
| PNUSAV000356 | 03/07/2018 | USA | Maryland | SAMN09917443 | PRJNA266293 |
| PNUSAV000398 | 08/08/2018 | USA | Oregon | SAMN10033026 | PRJNA266293 |
| PNUSAV000438 | 12/05/2018 | USA | Texas | SAMN10095497 | PRJNA266293 |
| PNUSAV000439 | 30/05/2018 | USA | Texas | SAMN10095496 | PRJNA266293 |
| PNUSAV000442 | 29/06/2018 | USA | Florida | SAMN10095504 | PRJNA266293 |
| PNUSAV000444 | 19/07/2018 | USA | Texas | SAMN10095502 | PRJNA266293 |
| PNUSAV000445 | 17/07/2018 | USA | Texas | SAMN10095501 | PRJNA266293 |
| PNUSAV000446 | 31/07/2018 | USA | Texas | SAMN10095500 | PRJNA266293 |
| PNUSAV000447 | 12/08/2018 | USA | Texas | SAMN10095499 | PRJNA266293 |
| PNUSAV000479 | 07/04/2018 | USA | Pennsylvania | SAMN10163151 | PRJNA266293 |
| PNUSAV000480 | 19/07/2018 | USA | Delaware | SAMN10163150 | PRJNA266293 |
| PNUSAV000481 | 28/08/2018 | USA | Ohio | SAMN10221805 | PRJNA266293 |
| PNUSAV000523 | 07/10/2018 | USA | NewYork | SAMN10391026 | PRJNA266293 |
| PNUSAV000525 | 14/10/2018 | USA | NewYork | SAMN10419344 | PRJNA266293 |
| PNUSAV000566 | 05/12/2018 | USA | NorthDakota | SAMN10794262 | PRJNA266293 |
| PNUSAV000580 | 20/01/2019 | USA | RhodeIsland | SAMN11159227 | PRJNA266293 |
| PNUSAV000950 | 12/08/2019 | USA | Massachusetts | SAMN12661289 | PRJNA266293 |
| PNUSAV000970 | 02/07/2019 | USA | California | SAMN12699060 | PRJNA266293 |
| PNUSAV001182 | 03/02/2020 | USA | Connecticut | SAMN14266007 | PRJNA266293 |
| PNUSAV001185 | 24/02/2020 | USA | Connecticut | SAMN14396416 | PRJNA266293 |
| PNUSAV001478 | 2020 | USA |  | SAMN17041481 | PRJNA266293 |
| PNUSAV001496 | 2021 | USA |  | SAMN18356821 | PRJNA266293 |
| PNUSAV001538 | 2021 | USA |  | SAMN19012332 | PRJNA266293 |
| PNUSAV001650 | 2021 | USA |  | SAMN20286048 | PRJNA266293 |
| PNUSAV001670 | 2021 | USA |  | SAMN20286032 | PRJNA266293 |
| PNUSAV001904 | 2021 | USA |  | SAMN20813321 | PRJNA266293 |
| PNUSAV002025 | 2021 | USA |  | SAMN22231286 | PRJNA266293 |
| PNUSAV002128 | 2021 | USA |  | SAMN22183831 | PRJNA266293 |
| PNUSAV002346 | 22/10/2021 | USA | Minnesota | SAMN23215746 | PRJNA266293 |
| PNUSAV002402 | 2021 | USA |  | SAMN23311458 | PRJNA266293 |
| PV170 | 06/05/2019 | Colombia | Cordoba | SAMN20804964 | PRJNA754786 |
| PV173 | 17/07/2019 | Colombia | Cordoba | SAMN20804965 | PRJNA754787 |
| PV278 | 30/07/2019 | Colombia | Bogota | SAMN20804967 | PRJNA754787 |
| PV280 | 24/07/2019 | Colombia | Cordoba | SAMN20804968 | PRJNA754787 |
| PV53 | 30/07/2018 | Colombia | Cordoba | SAMN20804969 | PRJNA754788 |
| PV85 | 16/10/2017 | Colombia | Cordoba | SAMN20804970 | PRJNA754789 |
| r46 | 2018 | China | Guangzhou | SAMN14411234 | PRJNA613630 |
| r75 | 2017 | China | Guangzhou | SAMN14411235 | PRJNA613630 |
| r79-2 | 2017 | China | Guangzhou | SAMN14411236 | PRJNA613630 |
| RIMD 2210633 | 1996 | Japan | Osaka | SAMD00058707 | PRJNA360 |
| S062 | 1998 | Singapore |  | SAMN02338919 | PRJNA215961 |
| S064 | 1998 | China | Taiwan | SAMN02338921 | PRJNA215961 |
| S066 | 1997 | China | Taiwan | SAMN02338923 | PRJNA215961 |
| S067 | 1997 | China | Taiwan | SAMN02338924 | PRJNA215961 |
| S068 | 1997 | China | Taiwan | SAMN02338925 | PRJNA215961 |
| S074 | 1997 | China | Taiwan | SAMN02338931 | PRJNA215961 |
| S075 | 1999 | China | Taiwan | SAMN02338932 | PRJNA215961 |
| S076 | 1999 | China | Taiwan | SAMN02338933 | PRJNA215961 |
| S077 | 1999 | China | Taiwan | SAMN02338934 | PRJNA215961 |
| S078 | 1999 | China | Taiwan | SAMN02338935 | PRJNA215961 |
| S083 | 1998 | Japan |  | SAMN02338939 | PRJNA215961 |
| S086 | 1999 | Thailand |  | SAMN02338940 | PRJNA215961 |
| S087 | 1998 | Singapore |  | SAMN02338941 | PRJNA215961 |
| S088 | 1998 | Singapore |  | SAMN02338942 | PRJNA215961 |
| S090 | 1999 | China | Taiwan | SAMN02338943 | PRJNA215961 |
| S091 | 1999 | India |  | SAMN02338944 | PRJNA215961 |
| S092 | 1996 | China | Taiwan | SAMN02338945 | PRJNA215961 |
| S094 | 1996 | Thailand |  | SAMN02338947 | PRJNA215961 |
| S133 | 2005 | China | Liaoning | SAMN02338981 | PRJNA215961 |
| S135 | 2003 | China | Guangxi | SAMN02338983 | PRJNA215961 |
| S136 | 2004 | China | Guangxi | SAMN02338984 | PRJNA215961 |
| S137 | 2005 | China | Guangxi | SAMN02338985 | PRJNA215961 |
| S138 | 2007 | China | Hubei | SAMN02338986 | PRJNA215961 |
| SH112 | 12/01/2019 | China | Shanghai | SAMN16205401 | PRJNA664306 |
| VIP4-0407 | 2008 | HongKong |  | SAMN02471133 | PRJNA222557 |
| VP100 | 2010 | China | Shenzhen | SAMN16783025 | PRJNA677930 |
| VP102 | 2011 | China | Shenzhen | SAMN16783027 | PRJNA677930 |
| VP111 | 2012 | China | Shenzhen | SAMN16783035 | PRJNA677930 |
| VP112 | 2011 | China | Shenzhen | SAMN16783036 | PRJNA677930 |
| VP121 | 2009 | China | Shenzhen | SAMN16783044 | PRJNA677930 |
| VP123 | 2009 | China | Shenzhen | SAMN16783046 | PRJNA677930 |
| VP137 | 2008 | China | Shenzhen | SAMN16783060 | PRJNA677930 |
| VP140 | 2009 | China | Shenzhen | SAMN16783063 | PRJNA677930 |
| VP141 | 2009 | China | Shenzhen | SAMN16783064 | PRJNA677930 |
| VP143 | 2009 | China | Shenzhen | SAMN16783066 | PRJNA677930 |
| VP146 | 2012 | China | Shenzhen | SAMN16783069 | PRJNA677930 |
| VP161168 | 12/08/2016 | China | Shanghai | SAMN09742583 | PRJNA483379 |
| VP161168 | 2016 | China |  | SAMEA8103029 | PRJEB39490 |
| VP161407 | 03/08/2016 | China | Shanghai | SAMN09742584 | PRJNA483379 |
| VP170 | 2012 | China | Shenzhen | SAMN16783090 | PRJNA677930 |
| VP176 | 2011 | China | Shenzhen | SAMN16783096 | PRJNA677930 |
| VP178 | 2011 | China | Shenzhen | SAMN16783097 | PRJNA677930 |
| VP185 | 2011 | China | Shenzhen | SAMN16783104 | PRJNA677930 |
| VP188 | 2012 | China | Shenzhen | SAMN16783107 | PRJNA677930 |
| VP204 | 2017 | China | Shenzhen | SAMN16783116 | PRJNA677930 |
| VP209 | 2016 | China | Shenzhen | SAMN16783118 | PRJNA677930 |
| VP218 | 2010 | China | Shenzhen | SAMN16783127 | PRJNA677930 |
| VP222 | 2010 | China | Shenzhen | SAMN16783131 | PRJNA677930 |
| VP230 | 2012 | China | Shenzhen | SAMN16783377 | PRJNA677930 |
| VP272 | 2017 | China | Shenzhen | SAMN16783173 | PRJNA677930 |
| VP285 | 2015 | China | Shenzhen | SAMN16783186 | PRJNA677930 |
| VP302 | 2015 | China | Shenzhen | SAMN16783203 | PRJNA677930 |
| VP341 | 2017 | China | Shenzhen | SAMN16783238 | PRJNA677930 |
| VP343 | 2017 | China | Shenzhen | SAMN16783240 | PRJNA677930 |
| VP354 | 2008 | China | Shenzhen | SAMN16783250 | PRJNA677930 |
| VP357 | 2008 | China | Shenzhen | SAMN16783253 | PRJNA677930 |
| VP374 | 2015 | China | Shenzhen | SAMN16783270 | PRJNA677930 |
| VP384 | 2013 | China | Shenzhen | SAMN16783279 | PRJNA677930 |
| VP394 | 2008 | China | Shenzhen | SAMN16783289 | PRJNA677930 |
| VP398 | 2016 | China | Shenzhen | SAMN16783293 | PRJNA677930 |
| VP399 | 2016 | China | Shenzhen | SAMN16783294 | PRJNA677930 |
| VP401 | 2012 | China | Shenzhen | SAMN16783296 | PRJNA677930 |
| VP402 | 2012 | China | Shenzhen | SAMN16783297 | PRJNA677930 |
| VP404 | 2012 | China | Shenzhen | SAMN16783299 | PRJNA677930 |
| VP405 | 2017 | China | Shenzhen | SAMN16783300 | PRJNA677930 |
| VP407 | 2017 | China | Shenzhen | SAMN16783389 | PRJNA677930 |
| VP440 | 2017 | China | Shenzhen | SAMN16783334 | PRJNA677930 |
| VP441 | 2017 | China | Shenzhen | SAMN16783335 | PRJNA677930 |
| VP443 | 2017 | China | Shenzhen | SAMN16783337 | PRJNA677930 |
| VP65 | 2013 | China | Shenzhen | SAMN16783356 | PRJNA677930 |
| VP72 | 2017 | China | Shenzhen | SAMN16783358 | PRJNA677930 |
| VP8 | 2016 | China | Shenzhen | SAMN16783345 | PRJNA677930 |
| VP85 | 2009 | China | Shenzhen | SAMN16783013 | PRJNA677930 |
| VP87 | 2009 | China | Shenzhen | SAMN16783015 | PRJNA677930 |
| VP88 | 2009 | China | Shenzhen | SAMN16783016 | PRJNA677930 |
| VP89 | 2009 | China | Shenzhen | SAMN16783017 | PRJNA677930 |
| VP90 | 2009 | China | Shenzhen | SAMN16783018 | PRJNA677930 |
| VP91 | 2009 | China | Shenzhen | SAMN16783019 | PRJNA677930 |
| VP96 | 2009 | China | Shenzhen | SAMN16783023 | PRJNA677930 |
| VPD14 | 2012 | China | Shanghai | SAMN09874427 | PRJNA487159 |
| VPF-1 | 19/07/2017 | Lebanon |  | SAMN08667565 | PRJNA437553 |
| VPF-2 | 02/08/2017 | Lebanon |  | SAMN08667566 | PRJNA437554 |
| VPF-3 | 02/08/2017 | Lebanon |  | SAMN08667567 | PRJNA437555 |
| VPF-4 | 02/08/2017 | Lebanon |  | SAMN08667585 | PRJNA437557 |
| VPF-5 | 16/08/2017 | Lebanon |  | SAMN08667654 | PRJNA437561 |
| VPF-6 | 18/09/2017 | Lebanon |  | SAMN08667664 | PRJNA437563 |
| VPF-7 | 18/10/2017 | Lebanon |  | SAMN08667669 | PRJNA437564 |
| MDOH-04-5M732 | 2004 | USA | Florida | SAMN13634001 | PRJNA531481 |

Supplementary Table 2- Full list of ecological and evolutionary features used within the model

|  | **Name** | **Description** |
| --- | --- | --- |
| **Evolutionary** | Total_Genes | Total number of genes identified in the isolate (core and accessory) |
|  | SNP_384 | Base present in isolate at position 384 |
|  | SNP_535 | Base present in isolate at position 535 |
|  | SNP_538 | Base present in isolate at position 538 |
|  | SNP_540 | Base present in isolate at position 540 |
|  | SNP_543 | Base present in isolate at position 543 |
|  | SNP_544 | Base present in isolate at position 544 |
|  | SNP_546 | Base present in isolate at position 546 |
|  | SNP_547 | Base present in isolate at position 547 |
|  | SNP_557 | Base present in isolate at position 557 |
|  | SNP_558 | Base present in isolate at position 558 |
|  | SNP_561 | Base present in isolate at position 561 |
|  | SNP_564 | Base present in isolate at position 564 |
|  | SNP_565 | Base present in isolate at position 565 |
|  | SNP_566 | Base present in isolate at position 566 |
|  | SNP_567 | Base present in isolate at position 567 |
|  | SNP_568 | Base present in isolate at position 568 |
|  | SNP_570 | Base present in isolate at position 570 |
|  | SNP_571 | Base present in isolate at position 571 |
|  | SNP_572 | Base present in isolate at position 572 |
|  | SNP_573 | Base present in isolate at position 573 |
|  | SNP_574 | Base present in isolate at position 574 |
|  | SNP_575 | Base present in isolate at position 575 |
|  | SNP_576 | Base present in isolate at position 576 |
|  | SNP_577 | Base present in isolate at position 577 |
|  | SNP_579 | Base present in isolate at position 579 |
|  | SNP_580 | Base present in isolate at position 580 |
|  | SNP_582 | Base present in isolate at position 582 |
|  | SNP_583 | Base present in isolate at position 583 |
|  | SNP_585 | Base present in isolate at position 585 |
|  | SNP_586 | Base present in isolate at position 586 |
|  | SNP_588 | Base present in isolate at position 588 |
|  | SNP_589 | Base present in isolate at position 589 |
|  | SNP_592 | Base present in isolate at position 592 |
|  | SNP_593 | Base present in isolate at position 593 |
|  | SNP_597 | Base present in isolate at position 597 |
|  | SNP_598 | Base present in isolate at position 598 |
|  | SNP_600 | Base present in isolate at position 600 |
|  | SNP_601 | Base present in isolate at position 601 |
|  | SNP_603 | Base present in isolate at position 603 |
|  | SNP_604 | Base present in isolate at position 604 |
|  | SNP_607 | Base present in isolate at position 607 |
|  | SNP_609 | Base present in isolate at position 609 |
|  | SNP_619 | Base present in isolate at position 619 |
|  | SNP_643 | Base present in isolate at position 643 |
|  | group_997 | Presence of Lactoylglutathione lyase in isolate |
|  | puuR | Presence of HTH-type transcriptional regulator (puuR) in isolate |
|  | rpoS_1 | Presence of RNA polymerase sigma factor (rpoS) in isolate |
|  | group_570 | Presence of Type IV pilus twitching motility protein (pilT) in isolate |
|  | group_2219 | Presence of Sodium:proton antiporter in isolate |
|  | aguC | Presence of N-carbamoylputrescine amidase (aguB) in isolate |
|  | aguB | Presence of Agmatine deiminase (aguC) in isolate |
|  | group_845 | Presence of DeoR family transcriptional regulator in isolate |
|  | group_569 | Presence of Type IV pilus twitching motility protein (pilT) in isolate |
|  | group_495 | Presence of Carbenicillin-hydrolyzing class A beta-lactamase CARB-23 in isolate |
|  | rnc | Presence of Ribonuclease III (rnc) in isolate |
|  | group_993 | Presence of tRNA 2-thiocytidine(32) synthetase (ttcA) in isolate |
|  | group_496 | Presence of Carbenicillin-hydrolyzing class A beta-lactamase CARB-23 in isolate |
|  | group_844 | Presence of DeoR family transcriptional regulator in isolate |
|  | ttcA | Presence of tRNA 2-thiocytidine(32) synthetase (ttcA) in isolate |
| **Ecological** | shellfish_imports_annual_tonnes | Annual shellfish imports into isolate country in isolate |
|  | SST_DJF_average | Sea Surface Temperature Average in coastal waters of isolate country over December-January-February of year of isolate discovery |
|  | SST_MAM_average | Sea Surface Temperature Average in coastal waters of isolate country over March-April-May of year of isolate discovery |
|  | SST_JJA_average | Sea Surface Temperature Average in coastal waters of isolate country over June-July-August of year of isolate discovery |
|  | SST_SON_average | Sea Surface Temperature Average in coastal waters of isolate country over September-October-November of year of isolate discovery |
|  | SST_DJF_min | Sea Surface Temperature Minimum in coastal waters of isolate country over December-January-February of year of isolate discovery |
|  | SST_MAM_min | Sea Surface Temperature Minimum in coastal waters of isolate country over March-April-May of year of isolate discovery |
|  | SST_JJA_min | Sea Surface Temperature Minimum in coastal waters of isolate country over June-July-August of year of isolate discovery |
|  | SST_SON_min | Sea Surface Temperature Minimum in coastal waters of isolate country over September-October-November of year of isolate discovery |
|  | SST_DJF_max | Sea Surface Temperature Maximum in coastal waters of isolate country over December-January-February of year of isolate discovery |
|  | SST_MAM_max | Sea Surface Temperature Maximum in coastal waters of isolate country over March-April-May of year of isolate discovery |
|  | SST_JJA_max | Sea Surface Temperature Maximum in coastal waters of isolate country over June-July-August of year of isolate discovery |
|  | SST_SON_max | Sea Surface Temperature Maximum in coastal waters of isolate country over September-October-November of year of isolate discovery |
|  | salinity_DJF_average | Salinity Average in coastal waters of isolate country over December-January-February of year of isolate discovery |
|  | salinity_MAM_average | Salinity Average in coastal waters of isolate country over March-April-May of year of isolate discovery |
|  | salinity_JJA_average | Salinity Average in coastal waters of isolate country over June-July-August of year of isolate discovery |
|  | salinity_SON_average | Salinity Average in coastal waters of isolate country over September-October-November of year of isolate discovery |
|  | salinity_DJF_min | Salinity Minimum in coastal waters of isolate country over December-January-February of year of isolate discovery |
|  | salinity_MAM_min | Salinity Minimum in coastal waters of isolate country over March-April-May of year of isolate discovery |
|  | salinity_JJA_min | Salinity Minimum in coastal waters of isolate country over June-July-August of year of isolate discovery |
|  | salinity_SON_min | Salinity Minimum in coastal waters of isolate country over September-October-November of year of isolate discovery |
|  | salinity_DJF_max | Salinity Maximum in coastal waters of isolate country over December-January-February of year of isolate discovery |
|  | salinity_MAM_max | Salinity Maximum in coastal waters of isolate country over March-April-May of year of isolate discovery |
|  | salinity_JJA_max | Salinity Maximum in coastal waters of isolate country over June-July-August of year of isolate discovery |
|  | salinity_SON_max | Salinity Maximum in coastal waters of isolate country over September-October-November of year of isolate discovery |
|  | SST_DJF_average_1yearlag | Sea Surface Temperature Average in coastal waters of isolate country over December-January-February of year previous to isolate discovery |
|  | SST_MAM_average_1yearlag | Sea Surface Temperature Average in coastal waters of isolate country over March-April-May of year previous to isolate discovery |
|  | SST_JJA_average_1yearlag | Sea Surface Temperature Average in coastal waters of isolate country over June-July-August of year previous to isolate discovery |
|  | SST_SON_average_1yearlag | Sea Surface Temperature Average in coastal waters of isolate country over September-October-November of year previous to isolate discovery |
|  | SST_DJF_max_1yearlag | Sea Surface Temperature Minimum in coastal waters of isolate country over December-January-February of year previous to isolate discovery |
|  | SST_MAM_max_1yearlag | Sea Surface Temperature Minimum in coastal waters of isolate country over March-April-May of year previous to isolate discovery |
|  | SST_JJA_max_1yearlag | Sea Surface Temperature Minimum in coastal waters of isolate country over June-July-August of year previous to isolate discovery |
|  | SST_SON_max_1yearlag | Sea Surface Temperature Minimum in coastal waters of isolate country over September-October-November of year previous to isolate discovery |
|  | SST_DJF_min_1yearlag | Sea Surface Temperature Maximum in coastal waters of isolate country over December-January-February of year previous to isolate discovery |
|  | SST_MAM_min_1yearlag | Sea Surface Temperature Maximum in coastal waters of isolate country over March-April-May of year previous to isolate discovery |
|  | SST_JJA_min_1yearlag | Sea Surface Temperature Maximum in coastal waters of isolate country over June-July-August of year previous to isolate discovery |
|  | SST_SON_min_1yearlag | Sea Surface Temperature Maximum in coastal waters of isolate country over September-October-November of year previous to isolate discovery |
|  | salinity_DJF_average_1yearlag | Salinity Average in coastal waters of isolate country over December-January-February of year previous to isolate discovery |
|  | salinity_MAM_average_1yearlag | Salinity Average in coastal waters of isolate country over March-April-May of year previous to isolate discovery |
|  | salinity_JJA_average_1yearlag | Salinity Average in coastal waters of isolate country over June-July-August of year previous to isolate discovery |
|  | salinitySON_average_1yearlag | Salinity Average in coastal waters of isolate country over September-October-November of year previous to isolate discovery |
|  | salinity_DJF_max_1yearlag | Salinity Minimum in coastal waters of isolate country over December-January-February of year previous to isolate discovery |
|  | salinity_MAM_max_1yearlag | Salinity Minimum in coastal waters of isolate country over March-April-May of year previous to isolate discovery |
|  | salinity_JJA_max_1yearlag | Salinity Minimum in coastal waters of isolate country over June-July-August of year previous to isolate discovery |
|  | salinity_SON_max_1yearlag | Salinity Minimum in coastal waters of isolate country over September-October-November of year previous to isolate discovery |
|  | salinity_DJF_min_1yearlag | Salinity Maximum in coastal waters of isolate country over December-January-February of year previous to isolate discovery |
|  | salinity_MAM_min_1yearlag | Salinity Maximum in coastal waters of isolate country over March-April-May of year previous to isolate discovery |
|  | salinity_JJA_min_1yearlag | Salinity Maximum in coastal waters of isolate country over June-July-August of year previous to isolate discovery |
|  | salinity_SON_min_1yearlag | Salinity Maximum in coastal waters of isolate country over September-October-November of year previous to isolate discovery |

Supplementary Table 3. SNP outliers detected with non-synonymous functional effects

| SNP Position | Predicted Effect | Variant Annotation | Ref Base | Alt Base | Base Count | | | | | |
| --- | --- | --- | --- | --- | --- | --- | --- | --- | --- | --- |
|  |  |  |  |  | A | C | T | G | N |  |
| SNP_384 | Medium | Missense | T | G | 0 | 0 | 307 | 2 | 0 |  |
| SNP_535 | Medium | Missense | C | N,T | 0 | 308 | 1 | 0 | 1 |  |
| SNP_538 | Medium | Missense | C | T | 0 | 309 | 1 | 0 | 0 |  |
| SNP_540 | Medium | Missense | C | T | 0 | 309 | 1 | 0 | 0 |  |
| SNP_543 | Medium | Missense | C | T | 0 | 309 | 1 | 0 | 0 |  |
| SNP_544 | Medium | Missense | A | G | 308 | 0 | 0 | 3 | 0 |  |
| SNP_546 | Medium | Missense | C | T | 0 | 296 | 14 | 0 | 0 |  |
| SNP_547 | Medium | Missense | G | A | 1 | 0 | 0 | 307 | 0 |  |
| SNP_557 | Medium | Missense | G | T | 0 | 0 | 1 | 307 | 0 |  |
| SNP_558 | Medium | Missense | T | C | 0 | 2 | 307 | 0 | 0 |  |
| SNP_561 | Medium | Missense | G | A | 1 | 0 | 0 | 307 | 0 |  |
| SNP_564 | Medium | Missense | C | T | 0 | 308 | 2 | 0 | 0 |  |
| SNP_565 | Medium | Missense | G | A | 1 | 0 | 0 | 307 | 0 |  |
| SNP_566 | Medium | Missense | G | C | 0 | 1 | 0 | 307 | 0 |  |
| SNP_567 | Medium | Missense | G | A | 1 | 0 | 0 | 307 | 0 |  |
| SNP_568 | Medium | Missense | A | T | 310 | 0 | 1 | 0 | 0 |  |
| SNP_570 | Medium | Missense | G | A | 1 | 0 | 0 | 307 | 0 |  |
| SNP_571 | Medium | Missense | G | A | 1 | 0 | 0 | 307 | 0 |  |
| SNP_572 | Medium | Missense | C | G | 0 | 309 | 0 | 1 | 0 |  |
| SNP_573 | Medium | Missense | A | G | 309 | 0 | 0 | 2 | 0 |  |
| SNP_574 | Medium | Missense | G | A | 1 | 0 | 0 | 307 | 0 |  |
| SNP_575 | Medium | Missense | C | A | 2 | 308 | 0 | 0 | 0 |  |
| SNP_576 | Medium | Missense | T | A | 1 | 0 | 308 | 0 | 0 |  |
| SNP_577 | Medium | Missense | C | G | 0 | 309 | 0 | 1 | 0 |  |
| SNP_579 | Medium | Missense | C | T | 0 | 308 | 2 | 0 | 0 |  |
| SNP_580 | Medium | Missense | G | T | 0 | 0 | 287 | 22 | 0 |  |
| SNP_582 | Medium | Missense | G | T | 0 | 0 | 1 | 307 | 0 |  |
| SNP_583 | Medium | Missense | T | C | 0 | 1 | 308 | 0 | 0 |  |
| SNP_585 | Medium | Missense | C | T | 0 | 310 | 0 | 0 | 0 |  |
| SNP_586 | Medium | Missense | G | A | 1 | 0 | 0 | 307 | 0 |  |
| SNP_588 | Medium | Missense | G | A | 1 | 0 | 0 | 307 | 0 |  |
| SNP_589 | Medium | Missense | G | A | 1 | 0 | 0 | 307 | 0 |  |
| SNP_592 | Medium | Missense | G | T | 0 | 0 | 1 | 307 | 0 |  |
| SNP_593 | Medium | Missense | A | G | 310 | 0 | 0 | 1 | 0 |  |
| SNP_597 | Medium | Missense | G | A | 3 | 0 | 0 | 305 | 0 |  |
| SNP_598 | Medium | Missense | C | T | 0 | 309 | 1 | 0 | 0 |  |
| SNP_600 | Medium | Missense | G | C | 0 | 1 | 0 | 307 | 0 |  |
| SNP_601 | Medium | Missense | T | A | 1 | 0 | 308 | 0 | 0 |  |
| SNP_603 | Medium | Missense | A | G | 310 | 0 | 0 | 1 | 0 |  |
| SNP_604 | Medium | Missense | A | G | 308 | 0 | 0 | 3 | 0 |  |
| SNP_607 | Medium | Missense | C | T | 0 | 309 | 1 | 0 | 0 |  |
| SNP_609 | Medium | Missense | G | A | 1 | 0 | 0 | 307 | 0 |  |
| SNP_619 | Medium | Missense | C | A | 0 | 310 | 0 | 0 | 0 |  |
| SNP_643 | Medium | Missense | C | T | 0 | 308 | 2 | 0 | 0 |  |

Supplementary Table 4. Error count analysis of eco-evolutionary models

| **Country-specific errors** | | | | | | | |
| --- | --- | --- | --- | --- | --- | --- | --- |
| Country | Populations | Temporal Evolution | Stages of expansion | Binary stages of expansion | Success | Total | % |
| Bangladesh | 0 | 0 | 2 | 0 | 1 | 3 | 30.00 |
| Canada | 0 | 0 | 1 | 0 | 0 | 1 | 20.00 |
| China | 1 | 0 | 7 | 2 | 5 | 15 | 8.11 |
| Colombia | 0 | 0 | 1 | 0 | 0 | 1 | 6.67 |
| India | 1 | 0 | 2 | 0 | 1 | 4 | 20.00 |
| Japan | 0 | 0 | 1 | 0 | 1 | 2 | 40.00 |
| Lebanon | 0 | 0 | 0 | 0 | 0 | 0 | 0.00 |
| Mexico | 1 | 1 | 0 | 0 | 1 | 3 | 60.00 |
| Peru | 1 | 2 | 5 | 4 | 4 | 16 | 17.78 |
| Singapore | 0 | 0 | 1 | 0 | 1 | 2 | 40.00 |
| Thailand | 0 | 0 | 2 | 0 | 1 | 3 | 20.00 |
| USA | 3 | 0 | 4 | 1 | 1 | 9 | 10.59 |
| Total | 7 | 3 | 26 | 7 | 16 |  |  |
| % | 7.78 | 3.33 | 28.89 | 7.78 | 17.78 |  |  |
| **Continent-specific errors** | | | | | | | |
| Continent | Populations | Temporal Evolution | Stages of expansion | Binary stages of expansion | Success | Total | % |
| Asia | 0 | 2 | 15 | 2 | 10 | 29 | 11.60 |
| North America | 1 | 4 | 4 | 1 | 2 | 12 | 12.63 |
| South America | 2 | 1 | 6 | 4 | 4 | 17 | 16.19 |
| Total | 3 | 7 | 25 | 7 | 16 |  |  |
| % | 3.33 | 7.78 | 27.78 | 7.78 | 17.78 |  |  |
| **Year-specific errors** | | | | | | | |
| Year | Populations | Temporal Evolution | Stages of expansion | Binary stages of expansion | Success | Total | % |
| 1996 | 0 | 0 | 3 | 0 | 1 | 4 | 20.00 |
| 1997 | 0 | 1 | 4 | 0 | 2 | 7 | 35.00 |
| 1998 | 0 | 2 | 4 | 0 | 4 | 10 | 28.57 |
| 1999 | 0 | 0 | 2 | 0 | 1 | 3 | 15.00 |
| 2000 | 0 | 0 | 0 | 0 | 0 | 0 | 0.00 |
| 2002 | 2 | 0 | 2 | 2 | 1 | 7 | 46.67 |
| 2003 | 0 | 0 | 1 | 1 | 0 | 2 | 40.00 |
| 2004 | 1 | 1 | 0 | 0 | 1 | 3 | 20.00 |
| 2005 | 0 | 0 | 0 | 0 | 1 | 1 | 20.00 |
| 2007 | 0 | 1 | 0 | 0 | 0 | 1 | 6.67 |
| 2008 | 0 | 0 | 0 | 0 | 0 | 0 | 0.00 |
| 2009 | 0 | 0 | 0 | 0 | 0 | 0 | 0.00 |
| 2010 | 0 | 0 | 0 | 0 | 0 | 0 | 0.00 |
| 2012 | 0 | 0 | 0 | 0 | 0 | 0 | 0.00 |
| 2013 | 0 | 0 | 0 | 0 | 0 | 0 | 0.00 |
| 2014 | 0 | 0 | 1 | 1 | 1 | 3 | 12.00 |
| 2015 | 0 | 1 | 1 | 1 | 2 | 5 | 25.00 |
| 2016 | 0 | 0 | 1 | 1 | 1 | 3 | 20.00 |
| 2017 | 0 | 1 | 0 | 0 | 0 | 1 | 1.82 |
| 2018 | 0 | 0 | 4 | 1 | 1 | 6 | 10.00 |
| 2019 | 0 | 0 | 0 | 0 | 0 | 0 | 0.00 |
| 2020 | 0 | 0 | 2 | 0 | 0 | 2 | 20.00 |
| Total | 3 | 7 | 25 | 7 | 16 |  |  |
| % | 3.33 | 7.78 | 27.78 | 7.78 | 17.78 |  |  |


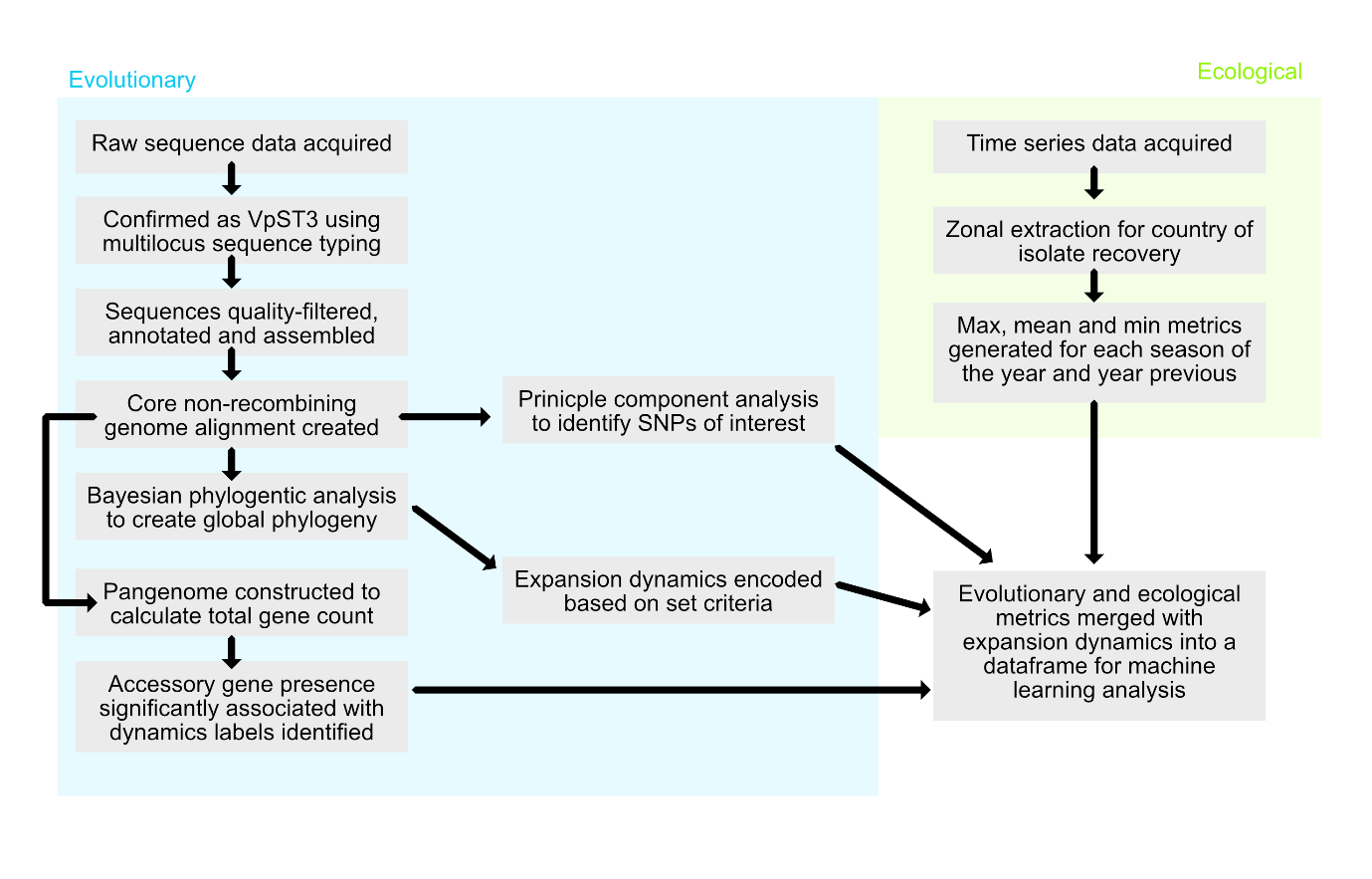


Supplementary Figure 1: Pre-processing protocol of evolutionary and ecological data into dataframe for machine learning analysis


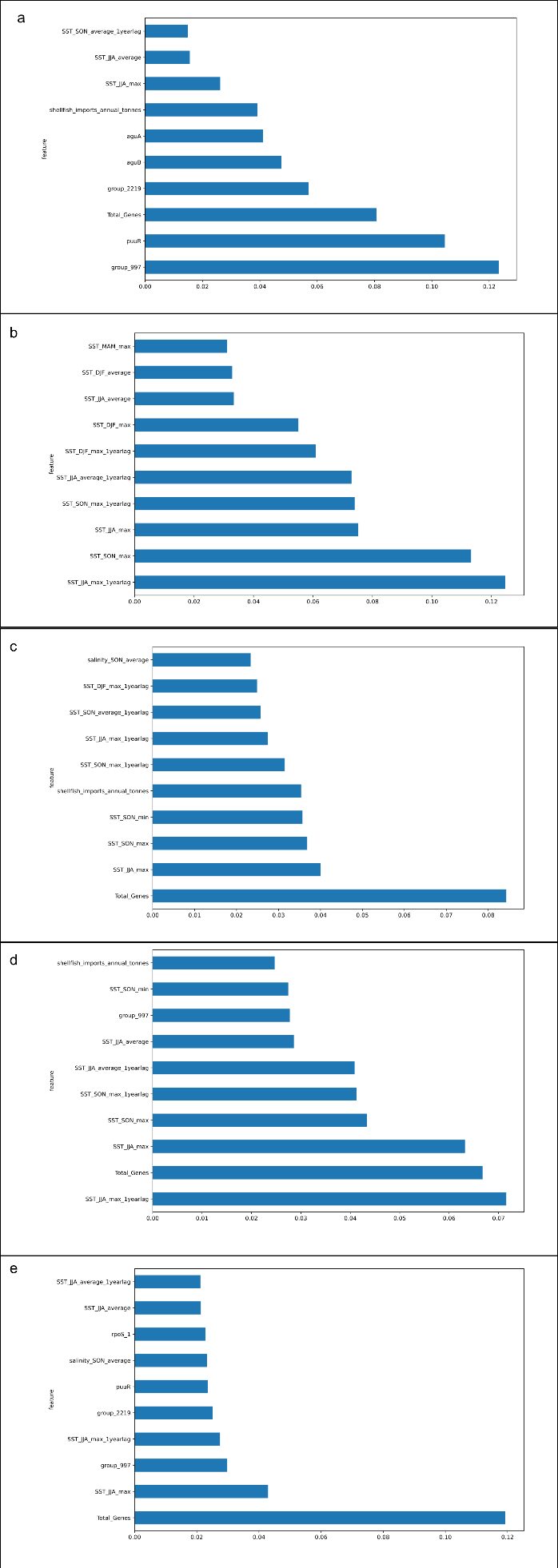


Supplementary Figure 2: Feature importance results for the 10 highest ranking features in eco-evolutionary models predicting a) populations, b) temporal evolution, c) stages of expansion, d) binary stages of expansion and e) success.


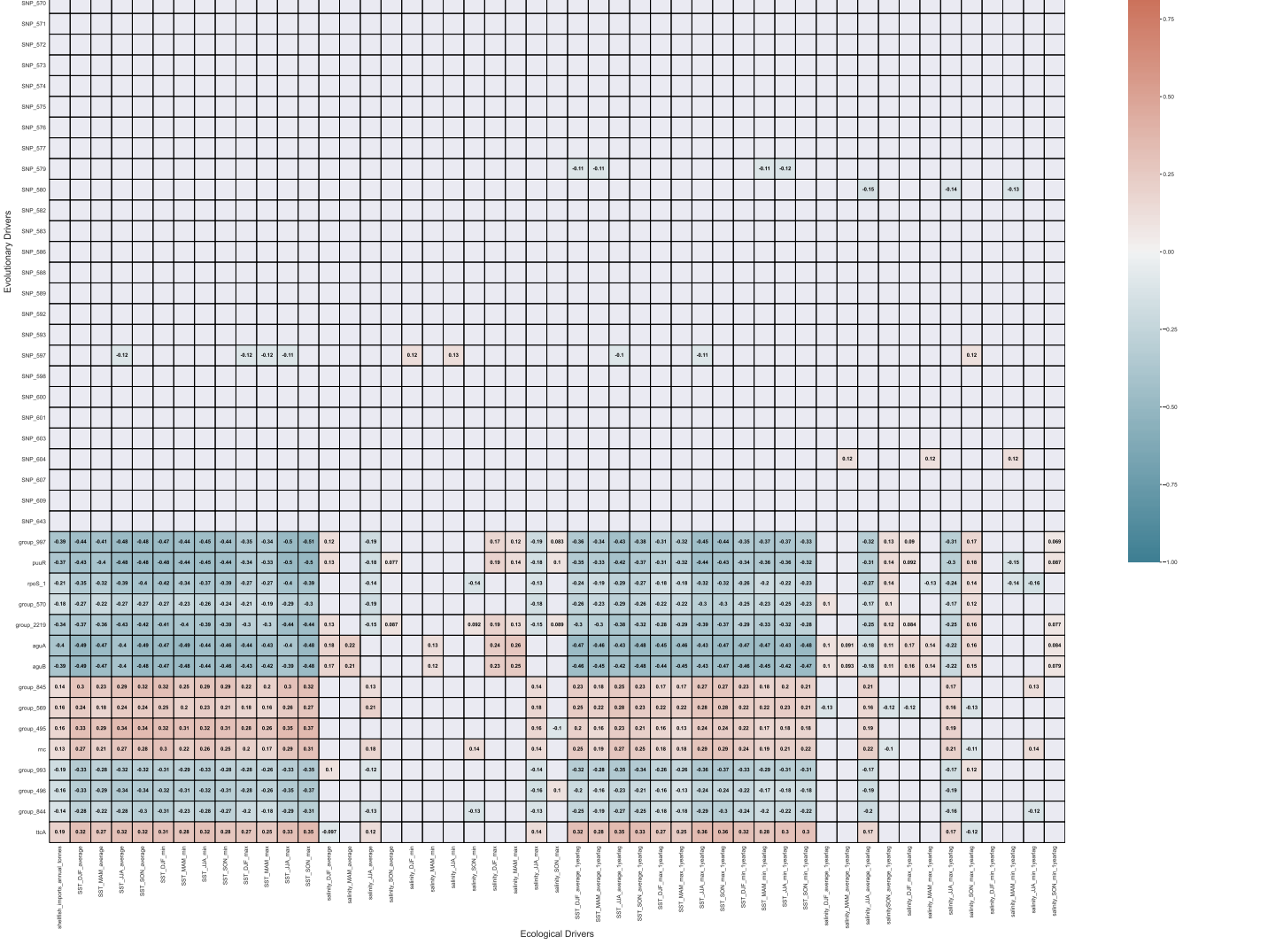


Supplementary Figure 3: Significant (p<0.05) Spearman’s Rank Correlation Coefficient between ecological and evolutionary features included in the model
